# Single alkyl phosphonate modification of the siRNA backbone in the seed region enhances specificity and therapeutic profile

**DOI:** 10.1093/nar/gkaf692

**Published:** 2025-07-30

**Authors:** Mehran Nikan, Qingfeng Li, Michael Tanowitz, Hongda Li, Sagar Damle, Marie Annoual, Rodrigo Galindo-Murillo, Audrey Low, Stephanie Klein, Clare Quirk, Guillermo Vasquez, W Brad Wan, Andrew T Watt, Michael T Migawa, Eric E Swayze, Thazha P Prakash

**Affiliations:** Ionis Pharmaceuticals Inc., 2855 Gazelle Ct, Carlsbad, CA 92010, United States; Ionis Pharmaceuticals Inc., 2855 Gazelle Ct, Carlsbad, CA 92010, United States; Ionis Pharmaceuticals Inc., 2855 Gazelle Ct, Carlsbad, CA 92010, United States; Ionis Pharmaceuticals Inc., 2855 Gazelle Ct, Carlsbad, CA 92010, United States; Ionis Pharmaceuticals Inc., 2855 Gazelle Ct, Carlsbad, CA 92010, United States; Ionis Pharmaceuticals Inc., 2855 Gazelle Ct, Carlsbad, CA 92010, United States; Ionis Pharmaceuticals Inc., 2855 Gazelle Ct, Carlsbad, CA 92010, United States; Ionis Pharmaceuticals Inc., 2855 Gazelle Ct, Carlsbad, CA 92010, United States; Ionis Pharmaceuticals Inc., 2855 Gazelle Ct, Carlsbad, CA 92010, United States; Ionis Pharmaceuticals Inc., 2855 Gazelle Ct, Carlsbad, CA 92010, United States; Ionis Pharmaceuticals Inc., 2855 Gazelle Ct, Carlsbad, CA 92010, United States; Ionis Pharmaceuticals Inc., 2855 Gazelle Ct, Carlsbad, CA 92010, United States; Ionis Pharmaceuticals Inc., 2855 Gazelle Ct, Carlsbad, CA 92010, United States; Ionis Pharmaceuticals Inc., 2855 Gazelle Ct, Carlsbad, CA 92010, United States; Ionis Pharmaceuticals Inc., 2855 Gazelle Ct, Carlsbad, CA 92010, United States; Ionis Pharmaceuticals Inc., 2855 Gazelle Ct, Carlsbad, CA 92010, United States

## Abstract

We evaluated the effect of alkyl phosphonate linkages in enhancing the specificity and therapeutic profile of siRNA when incorporated into the seed region. siRNAs modified with a single alkyl phosphonate linkage demonstrated enhanced specificity and therapeutic profile compared to the parent siRNA. We found that these modifications are most effective when positioned at the internucleotide linkages 6–7 from the 5′-end of the guide strand. Our findings reveal that siRNAs with this modification maintain robust on-target activity both *in vitro* and *in vivo*. Importantly, differential gene expression (DGE) analysis showed a significant reduction in off-target effects across *in vitro* and *in vivo*, leading to an improved therapeutic profile. We also demonstrate enhanced safety in mice, as evidenced by reduced ALT/AST elevation and the absence of histopathological changes. This novel chemical approach to siRNA design provides impetus to advancing RNA interference-based treatments for various diseases.

## Graphical Abstract

**Graphical Abstract:**
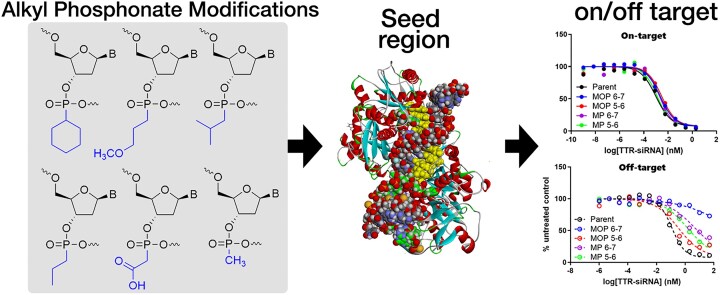

## Introduction

Exogenous siRNAs do not always exhibit specificity, potentially resulting in unintended consequences on the expression of non-targeted genes, referred to as off-target effects. The first reported instance of siRNA off-targeting dates back to 2003 when Jackson et al. observed differential expression in a significant number of genes following siRNA treatment using whole genome microarray analysis [1]. Although no clear pattern for predicting off-targeting emerged, the authors correctly speculated that the 5′-end of the guide strand plays a crucial role in transcript modulation. This finding aligned with early observations in the microRNA (miRNA) field, suggesting that miRNAs regulate gene functions by binding to the 3′-UTRs of transcripts [2], utilizing nucleotides 2-8 from the 5′-end, commonly referred to as the seed region [3]. Subsequent studies provided more insights into the role of the seed region of siRNAs and these off-targeting pathways, now recognized as the miRNA-like effect [4–6]. It was also observed that the miRNA-like effect is closely linked to the thermodynamic stability of the seed-mRNA duplex, determined by the melting temperature and standard free energy changes [7].

Since the sequence determinant of off-targeting is known, and some of the thermodynamic parameters governing the off-targeting can be calculated, extensive research has been directed towards the advancement of bioinformatic tools [8–10]. These tools aim to anticipate potential off-targets by identifying sites with 8mer, 7mer, and 6mer sequences aligning with the seed region of the siRNA. Although the seed region plays a pivotal role in off-targeting, the inclusion of non-seed matches further complicates prediction models by complementing the original bindings [11]. The prediction models for siRNAs face additional hurdles. The prevalence of perfect or partial seed matches across the transcriptome poses a substantial obstacle and discerning which among them might exert a regulatory influence adds additional complexities. Despite the ongoing progress in this domain, supported by advancements in the field of miRNA research [12], these computational tools cannot entirely predict the specificity of siRNAs with certainty or replace experimental methodologies.

While miRNA-like effects represent a prominent form of siRNA off-targeting, additional pathways also play a role. These alternative off-targeting pathways are not all well-characterized, but they may involve triggering an immune response [13], disrupting mRNA secondary structures, blocking miRNA pathways, or interacting with intracellular proteins. The passenger strand is recognized as another contributor to off-target effects [14]. Some of these alternative off-targeting pathways can be effectively suppressed through chemical modifications. For instance, modifications at the 2′-position of the ribose are known to alleviate immune response [15, 16], and modifications at the 5′-end of the passenger strand can eliminate off-targeting induced by it [14, 17].

Chemical modifications which modulate seed affinity have demonstrated effectiveness in mitigating miRNA-like effects. An early example of chemical approaches was illustrated by Jackson et al., who showed that 2′-O-methyl modification in the guide strand, especially at position 2, reduces unintended target affinity [18]. Following this, Ui-Tei et al. demonstrated that the substitution of the seed region of siRNA with DNA effectively alleviates seed-specific off-target effects [19]. In search of more effective modifications, Bramsen et al. conducted a comprehensive screen of 10 different chemical modifications, systematically incorporating them throughout the seed region at various positions. Their study identified unlocked nucleic acid (UNA) as the most effective modification for enhancing siRNA specificity. When introduced into the seed region, UNA demonstrated a significant reduction in miRNA-like activity in a position-dependent manner without compromising on-target silencing efficacy [20]. Following this seminal work, various other modifications have been introduced in the literature to tackle off-target activity, primarily utilizing sugar or nucleobase modifications [21–24]. Schlegel et al. recently reported an elegant illustration of this approach, demonstrating that (S)-GNA in the seed region of the siRNA could effectively reduce miRNA-like off-targeting in an *in vivo* setting [25–28].

It is also well established that duplex flexibility and binding dynamics can be modulated through backbone modifications [29–31]. However, only a limited number of studies have explored the impact of these modifications on reducing off-target effects and improving siRNA specificity [27, 32], suggesting that this area remains underexplored. In this study, we demonstrate that a single alkyl phosphonate modification in the seed region enhances the therapeutic profile of siRNAs both *in vitro* and *in vivo*, paving the way for their clinical applications.

## Material and methods

### General

All commercial starting materials, reagents and solvents were purchased from Sigma-Aldrich and were used without further purification. Anhydrous solvents and reagents were purchased from commercial sources and used without further purification. Thin layer chromatography analyses were performed on TLC Silicagel 60 F_254_ plates and column chromatographic purifications were performed on Biotage Isolera One using Sfar prepacked silica columns. All reactions were performed under argon atmosphere unless otherwise stated. Deuterated solvents were purchased from Cambridge Isotope Laboratories. ^1^H, ^13^C and ^32^P NMR spectra were recorded on Bruker Avance 300 spectrometer using the residual solvent signal as the reference. LC-MS spectra were recorded on an Agilent 1260 Infinity II coupled with an Agilent 6130 single quadrupole mass spectrometer.

### Synthesis of alkyl phosphonamidites

Methyl phosphonamidites were obtained from Glen Research. Isobutyl, propyl, and MOP phosphonamidites were synthesized using alkyl-bis(diisopropylamino)phosphine **3b-d** as the starting material. For isobutyl and propyl derivatives, commercial Grignard reagents (2 M solutions in diethyl ether) were employed, while MOP derivatives were prepared with synthesized Grignard reagents following a reported procedure [33]. Cyclohexyl phosphonamidites were synthesized from cyclohexyldichlorophosphine **5**, as detailed below.

### General method for the synthesis of alkyl-bis(diisopropylamino)phosphine 3b-d


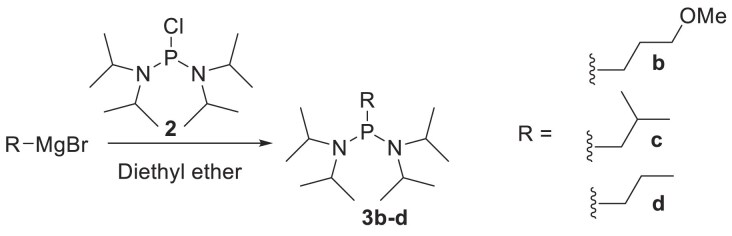


In a typical reaction, bis(diisopropylamino)chlorophosphine **2** (5 g, 18.74 mmol, 1 equiv.) was placed in a 250 mL flask and dried over P_2_O_5_ overnight under vacuum. The dried compound was then suspended in anhydrous Et_2_O (60 mL), and the reaction mixture was cooled to 0°C in an ice bath with stirring under nitrogen. The Grignard reagent (either commercially available or synthesized; 20 mmol, 1.1 equiv.) was added via a syringe (for iBu and Prop), or via a cannula (for MOP). The reaction was allowed to warm to room temperature and stirred for 1 h, with progress monitored by ^31^P NMR. The reaction mixture was filtered through a celite plug, and the solids were rinsed with Et_2_O. The combined filtrates were concentrated using a rotary evaporator. The residue was suspended in anhydrous acetonitrile (40 mL) and transferred to a separatory funnel for extraction with hexanes (2 × 100 mL). The hexanes layer was washed with acetonitrile (2 × 30 mL), then passed through a cotton plug to remove particulates. The filtrate was concentrated on a rotary evaporator and briefly dried under high vacuum to yield the phosphine reagent as an oil. This crude reagent was used in the subsequent step without further purification.

**3b (MOP):** Previously synthesized and characterized [33]. **3c (iBu):** 3.9 g (72%), ^1^H NMR (300 MHz, acetone-d6) δ 3.19-3.43 (m, 4H), 1.36-1.63 (m, 3H), 1.07 (d, J = 6.73 Hz, 12H), 0.96 (d, J = 6.64 Hz, 12H), 0.89 (d, J = 6.19 Hz, 6H), ^31^P NMR (121 MHz, acetone-d_6_) δ 43.72. **3d (Prop):** 3.6 g (70%), ^1^H NMR (300 MHz, CDCl_3_) δ 3.39 (quint, J = 6.66, 10.18 Hz, 4H), 1.54-1.69 (m, 2H), 1.32-1.51 (m, 2H), 1.18 (d, J = 6.73 Hz, 12H), 1.08 (d, J = 6.64 Hz, 12H), 1.00 (t, J = 7.23 Hz, 3H), ^31^P NMR (121 MHz, CDCl_3_) δ 47.10.

### General method for the synthesis of alkyl phosphonamidites 4b-d


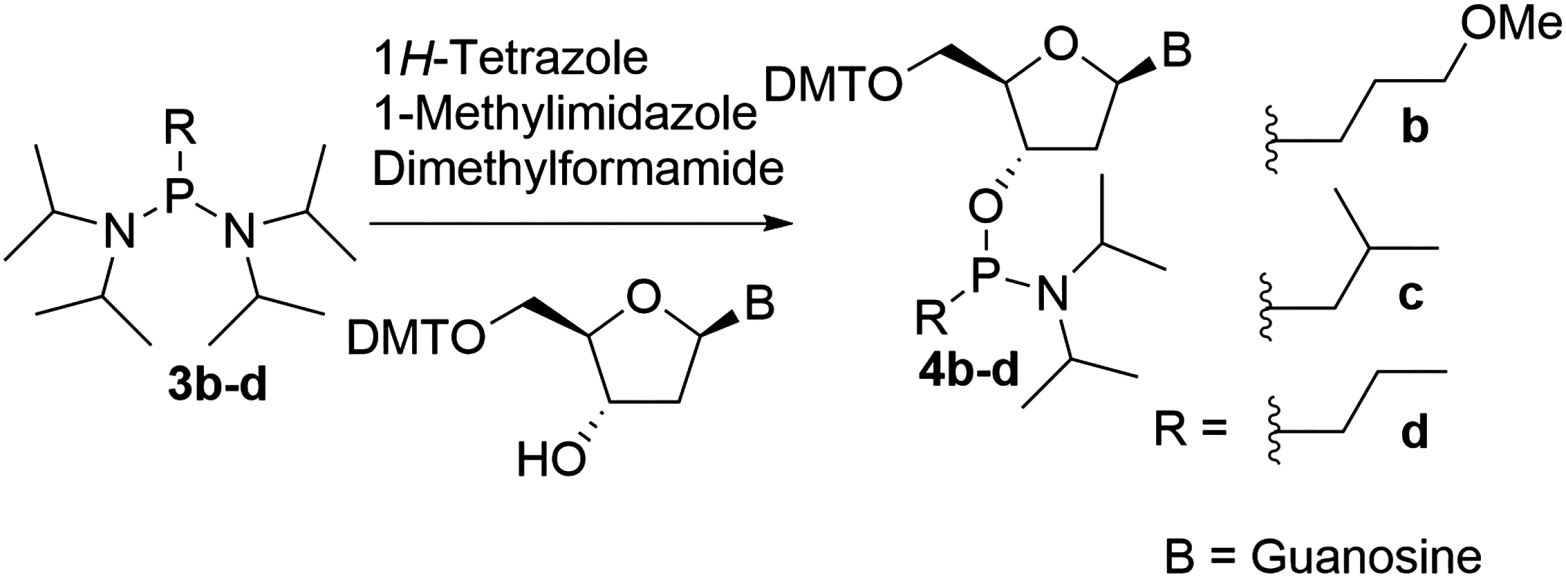


N²-isobutyryl-2′-deoxy-5′-dimethoxytrityl-guanosine (2.0 g, 3.04 mmol, 1 equiv.) was dissolved in anhydrous dimethylformamide (0.1 M) followed by addition of 1*H*-tetrazole (170 mg, 2.43 mmol, 0.8 equiv.) and 1-methyl imidazole (109 mg, 1.34 mol, 0.44 equiv.). Next, the phosphine reagent **3b-d** (4.56 mmol, 1.5 equiv.) was added, and the reaction was allowed to stir at room temperature for 24 h under nitrogen. The progress of reaction was monitored by TLC. After 24 hours, the reaction was terminated, and the solvent was removed with a rotary evaporator, keeping the bath temperature below 35°C. The residue was diluted with ethyl acetate (100 mL) and transferred to a separatory funnel. The organic layer was washed with water (200 ml), followed by saturated aqueous NaHCO_3_ (200 mL), and finally with saturated NaCl (100 mL). The organic layers were pooled, dried over MgSO_4_, filtered and concentrated. The residue was purified by silica gel chromatography to yield the desired phosphonamidites **4b-d** and the unreacted starting material was recovered.

**4b (MOP):** Previously synthesized and characterized [33]. **4c (iBu):** The protected nucleoside (4 g, 6.25 mmol) was converted to the product (3.7 g, 71%), ^1^H NMR (300 MHz, acetone-d6) δ 12.07 (br s, 1H), 10.32 (br s, 1H), 7.95 (d, J = 1.44 Hz, 1H), 7.18–7.54 (m, 9H), 6.78–6.92 (m, 4H), 6.26 (ddd, J = 1.84, 5.95, 7.92 Hz, 1H), 4.44–4.82 (m, 1H), 4.20 (br d, J = 2.42 Hz, 1H), 3.79 (s, 6H), 3.56 (br d, J = 5.56 Hz, 2H), 3.21–3.40 (m, 2H), 2.77–3.00 (m, 2H), 2.39–2.68 (m, 1H), 1.64–1.91 (m, 1H), 1.44–1.63 (m, 2H), 1.08–1.27 (m, 18H), 0.99–1.07 (m, 6H), ^31^P NMR (121 MHz, CDCl_3_) δ 125.4, 126.5. ESI-MS m/z: [M-H]^−^, found 825.4. **4d (Prop):** The protected nucleoside (1 g, 1.56 mmol) was converted to the product (0.9 g, 70%), ^1^H NMR (300 MHz, acetone-d6) δ 12.06 (br s, 1H), 10.35 (br s, 1H), 7.95 (d, J = 3.23 Hz, 1H), 7.16–7.56 (m, 9H), 6.74–6.95 (m, 4H), 6.00–6.37 (m, 1H), 4.45–4.82 (m, 1H), 4.14–4.33 (m, 1H), 3.79 (s, 6H), 3.46–3.68 (m, 2H), 3.25–3.43 (m, 2H), 2.76–2.99 (m, 2H), 2.38–2.68 (m, 1H), 1.34–1.72 (m, 4H), 1.08–1.31 (m, 18H), 1.03 (dt, J = 3.81, 7.20 Hz, 3H), ^31^P NMR (121 MHz, acetone-d6) δ 125.72, 126.55. ESI-MS m/z: [M-H]^−^, found 811.4.

### Synthesis of cyclohexyl phosphonamidite 4a


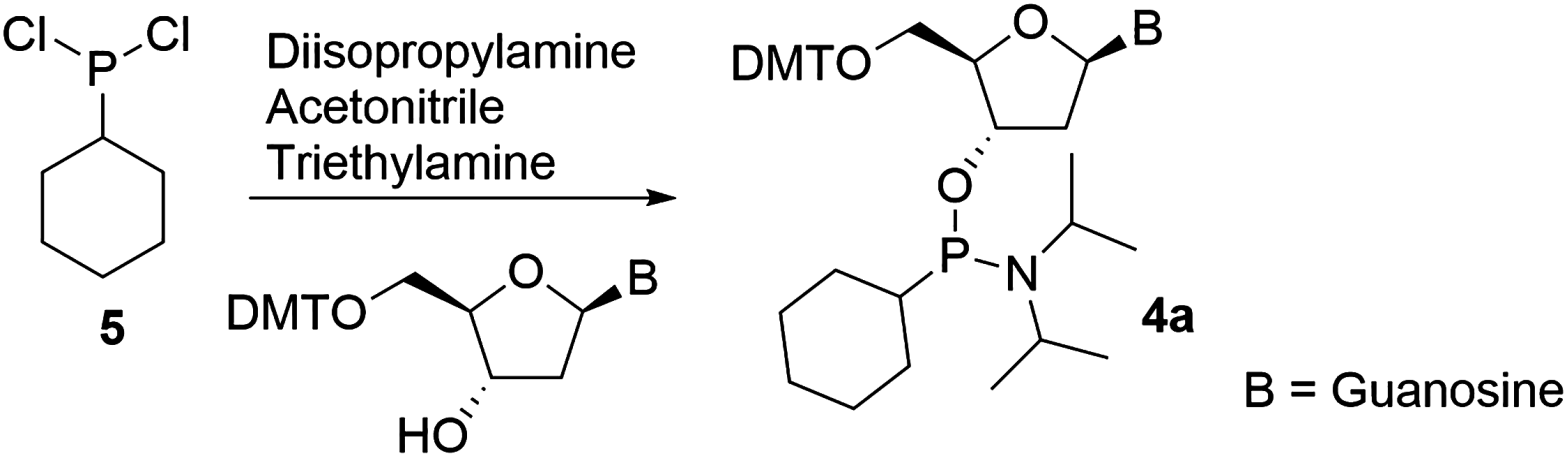


*N*,*N*-diisopropylamine (0.379 g, 3.75 mmol) was dissolved in anhydrous acetonitrile (10 mL) and cooled to -20°C. Cyclohexyldichlorophosphine (0.318 g, 1.72 mmol) was then added, and the mixture stirred at −20°C for 20–40 min. In a separate vessel, N²-isobutyryl-2′-deoxy-5′-dimethoxytrityl-guanosine (1.0 g, 1.56 mmol) was dissolved in anhydrous dichloromethane (10 mL) along with triethylamine (0.221 g, 2.19 mmol). This solution was added to the reaction mixture, which was then allowed to warm to room temperature and stirred overnight under nitrogen. After concentration, the crude product was purified by silica gel chromatography using 1:1 hexanes- ethyl acetate as eluent, yielding 250 mg of the desired product (18% yield).

**4a (cHex):** ^1^H NMR (300 MHz, CDCl_3_) δ 11.89 (br s, 1H), 7.75–7.82 (m, 1H), 7.49 (td, J = 1.66, 6.37 Hz, 2H), 7.32–7.43 (m, 4H), 7.14–7.30 (m, 3H), 6.73–6.82 (m, 4H), 6.05–6.26 (m, 1H), 4.60–4.79 (m, 1H), 4.19–4.33 (m, 1H), 3.74–3.82 (m, 6H), 3.27–3.71 (m, 4H), 2.92–3.23 (m, 2H), 1.63–1.93 (m, 8H), 0.94–1.19 (m, 18H), 0.75–0.90 (m, 3H), ^31^P NMR (121 MHz, CDCl_3_) δ 131.0, 131.1. ESI-MS m/z: [M-H]^−^, found 851.4.

### Synthesis of 2′-fluoro and 2′-OMe MOP phosphonamidites 6b and 7b

The 2′-F and 2′-OMe MOP phosphonamidites (**6b** and **7b**) were synthesized by applying the general method for preparing alkyl phosphonamidites (**4b-d**), starting from the corresponding 2′-F or 2′-OMe nucleosides.


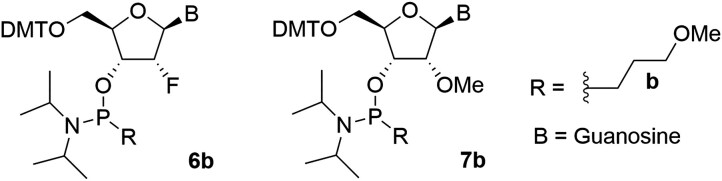


**6b (2′-F, MOP):** The protected nucleoside (2 g, 3.04 mmol) was converted to the product (1.3 g, 50%),

^1^H NMR (300 MHz, acetone-d6) δ 12.10 (br s, 1H), 10.40 (br s, 1H), 8.01 (d, J = 13.82 Hz, 1H), 7.44–7.57 (m, 2H), 7.21–7.43 (m, 7H), 6.77–6.96 (m, 4H), 6.02–6.32 (m, 1H), 6.18 (ddd, J = 2.87, 10.46, 15.48 Hz, 1H), 5.29–5.88 (m, 1H), 5.26–5.89 (m, 1H), 4.40–4.91 (m, 1H), 4.18–4.40 (m, 1H), 3.80 (d, J = 1.80 Hz, 6H), 3.31–3.66 (m, 6H), 3.25–3.31 (m, 3H), 2.75–2.99 (m, 2H), 1.41–1.83 (m, 4H), 0.96–1.28 (m, 18H), ^31^P NMR (121 MHz, CDCl_3_) δ 149.7 (d), 15.6 (d). ESI-MS m/z: [M-H]^−^, found 859.4. **7b (2′-OMe, MOP):** The protected nucleoside (6 g, 9.0 mmol) was converted to the product (4.1 g, 52%), ^1^H NMR (300 MHz, acetone-d6) δ 12.04 (br s, 1H), 10.24 (br s, 1H), 7.93–8.02 (m, 1H), 7.46–7.53 (m, 2H), 7.19–7.44 (m, 7H), 6.83–6.94 (m, 4H), 5.85–6.01 (m, 1H), 4.59 (br d, J = 7.18 Hz, 1H), 4.40–4.57 (m, 1H), 4.20–4.36 (m, 1H), 3.80 (s, 6H), 3.51–3.68 (m, 2H), 3.32–3.51 (m, 7H), 3.29 (s, 3H), 2.71–2.90 (m, 2H), 1.50–1.96 (m, 4H), 1.12–1.22 (m, 18H), ^31^P NMR (121 MHz, CDCl_3_) δ 132.91. ESI-MS m/z: [M-H]^−^, found 871.4.

### Synthesis of *Sp* and *Rp* diastereomers 10 and 11

The synthesis of *Sp* and *Rp* diastereomers **10** and **11** was performed as outlined in Scheme 2. 3′-*O*-tert-butyldimethylsilyl-N^6^-benzoyl-2′-*O*-methyl-adenosine **8** was prepared by 3′-*O*-silylation of the corresponding 5′-*O*-dimethoxytrityl-N^6^-benzoyl-2′-*O*-methyl-nucleoside, followed by 5′-*O*-detritylation with benzenesulfonic acid as previously described [34].

For the preparation of the *Rp* and *Sp* isomers, compound **8** (1.45 g, 2.9 mmol, 1.0 equiv.) and phosphonamidite **4c** (2.40 g, 2.9 mmol, 1.0 equiv.) were dissolved in 36 mL anhydrous acetonitrile under nitrogen. 1*H*-tetrazole (1.421 g, 20.3 mmol, 7.0 equiv.) was added, and the reaction mixture was stirred at room temperature for 15 min. Subsequently, tert-butyl hydroperoxide (70 wt. % in water, 2 mL) was added, and stirring was continued for an additional 15 min. Reaction progress was monitored by TLC (100% ethyl acetate, 1% triethylamine), which confirmed completion. The reaction was quenched by adding 4 mL of saturated sodium bisulfite solution. The solvent was removed under reduced pressure, and the residue was suspended in dichloromethane. The suspension was washed sequentially with saturated sodium bicarbonate solution, water, and brine. The organic layer was dried over anhydrous sodium sulfate, filtered, and concentrated under reduced pressure. The crude product was purified by column chromatography on silica gel, eluting with a gradient of 0–5% methanol in ethyl acetate containing 1% triethylamine. Poor resolution was observed between the *Rp* (faster eluting) and *Sp* (slower eluting) isomers **9a-b**. The combined *Rp* and *Sp* mixture was subjected to TBS deprotection.

To the crude **9a-b** (2 g, *Rp*/*Sp* mixture, 1.62 mmol, 1 equiv.), 12 mL anhydrous THF and 0.56 mL (4 mmol, 2.5 equiv.) triethylamine were added. Then, 1.33 mL (8 mmol, 5 equiv.) triethylamine trihydrofluoride (TREAT-HF) was added dropwise. The mixture was stirred at room temperature overnight and monitored by LC-MS and TLC (9:1 ethyl acetate–methanol) to confirm completion. The reaction was quenched by adding 1.4 mL of triethylamine, and the solvent was evaporated to dryness. The pure *Sp* and *Rp* isomers **10** and **11** were then separated by reverse-phase chromatography using a gradient of 0–90% acetonitrile in water over 90 min.

**10:** (500 mg, 15% overall yield, two steps), ^1^H NMR (300 MHz, acetone-d6) δ 11.94 (s, 1H), 10.70 (s, 1H), 9.74-10.15 (m, 1H), 8.68 (s, 1H), 8.57 (s, 1H), 8.12–8.25 (m, 2H), 7.89 (s, 1H), 7.51–7.71 (m, 3H), 7.12–7.45 (m, 10H), 6.69–6.86 (m, 4H), 6.26 (d, J = 3.68 Hz, 1H), 6.11–6.22 (m, 1H), 5.59–5.74 (m, 1H), 4.74 (s, 1H), 4.60–4.69 (m, 1H), 4.44 (dd, J = 4.49, 6.91 Hz, 2H), 4.37–4.55 (m, 1H), 4.23–4.34 (m, 1H), 4.15 (q, J = 4.31 Hz, 1H), 3.77 (s, 6H), 3.53 (s, 3H), 3.24–3.38 (m, 2H), 2.93–3.10 (m, 1H), 2.73–2.90 (m, 4H), 2.54–2.71 (m, 1H), 1.89–2.03 (m, 1H), 1.61–1.77 (m, 2H), 1.19 (dd, J = 2.20, 6.87 Hz, 6H), 0.98 (dd, J = 0.67, 6.60 Hz, 6H), ^31^P NMR (121 MHz, acetone-d6) δ 32.59. ESI-MS m/z: [M-H]^−^, found 1225.4. **11:** (660 mg, 20% overall yield, two steps), ^1^H NMR (300 MHz, acetone-d6) δ 11.97 (s, 1H), 10.61 (s, 1H), 9.98 (br s, 1H), 8.70 (s, 1H), 8.53 (s, 1H), 8.11 (d, J = 7.36 Hz, 2H), 7.92 (s, 1H), 7.61–7.71 (m, 1H), 7.51–7.61 (m, 2H), 7.34–7.46 (m, 2H), 7.31 (br d, J = 2.33 Hz, 1H), 7.11–7.72 (m, 8H), 6.81 (dd, J = 1.62, 8.89 Hz, 4H), 6.26 (d, J = 3.68 Hz, 1H), 6.21 (t, J = 6.64 Hz, 1H), 5.39–5.55 (m, 1H), 4.60–4.75 (m, 2H), 4.18–4.49 (m, 5H), 3.77 (s, 6H), 3.55 (s, 3H), 3.33 (dd, J = 2.69, 4.22 Hz, 1H), 3.29–3.39 (m, 1H), 3.04 (td, J = 6.65, 13.71 Hz, 1H), 2.75–2.94 (m, 4H), 2.67 (ddd, J = 3.64, 6.10, 13.78 Hz, 1H), 1.76–1.88 (m, 2H), 1.22 (dd, J = 6.91, 7.99 Hz, 6H), 0.99–1.06 (m, 6H), ^31^P NMR (121 MHz, acetone-d6) δ 32.70. ESI-MS m/z: [M-H]^−^, found 1225.4.

### General method for the synthesis of phosphoramidites 12 and 13

Compounds **10** or **11** (490 mg, 0.435 mmol, 1.0 equiv.) was dissolved in 5 mL anhydrous dichloromethane under a nitrogen atmosphere. Anhydrous triethylamine (243 μL, 1.74 mmol, 4.0 equiv.) was added, and the solution was stirred for 2 min at room temperature. 2-Cyanoethyl *N*,*N*-diisopropylchlorophosphoramidite (153 μL, 0.653 mmol, 1.5 equiv.) was then added dropwise. The reaction mixture was stirred at room temperature for 2 h under nitrogen. Progress of the reaction was monitored by TLC using dichloromethane/ methanol (95:5) with 1% triethylamine as the eluent. Upon completion, the solvent was removed under reduced pressure to yield a light-yellow oil. The residue was partitioned between dichloromethane (200 mL) and saturated sodium bicarbonate solution (50 mL). The organic layer was washed sequentially with water (50 mL) and brine (50 mL), dried over anhydrous magnesium sulfate, filtered, and concentrated under reduced pressure. The crude product was precipitated from hexanes and used directly for subsequent coupling reactions without further purification.

**12:** (crude), ^31^P NMR (121 MHz, acetone-d6) δ 150.6, 149.8, 32.5. ESI-MS m/z: [M-H]^−^, found 1325.5. **13:** (crude), ^31^P NMR (121 MHz, acetone-d6) δ 150.4, 149.7, 32.5. ESI-MS m/z: [M-H]^−^, found 1325.5.

### Oligonucleotide synthesis

Guide strands were synthesized on an Applied Biosystems 394 DNA/RNA Synthesizer at 2 μmol scale on VIMAD UnyLinker support (200 μmol/g). Standard solid-phase synthesis protocols were followed, including deblocking with 3% dichloroacetic acid in dichloromethane, activation using 4,5-dicyanoimidazole (1 M) and N-methylimidazole (0.1 M) in acetonitrile, capping with 10% acetic anhydride in tetrahydrofuran (THF) and 10% N-methylimidazole in THF/pyridine, and thiolation using xanthane hydride (0.1 M) in pyridine: acetonitrile (3:2 v/v). Phosphoramidites were prepared at 0.1 M in acetonitrile with 6-min coupling times, except for 5′-POM-(*E*)-Vinylphosphonate (VP)-2′-MOE-T phosphoramidite, which was prepared at a concentration of 0.15 M. Post-synthesis, oligonucleotides were cleaved and deprotected in 10% diethylamine/90% (9:1 NH_4_OH-EtOH) for 36 h at room temperature. After filtration, the crude mixture was purified by ion-pair reversed-phase HPLC on an XBridge Prep C18 column (5 μm, 19 × 250 mm) using a 1–90% acetonitrile gradient over 60 min in 5 mM tetrabutylammonium acetate (TBAA). Pure fractions were pooled and further purified by strong anion-exchange chromatography on SOURCE 30Q resin using 100 mM ammonium acetate (Buffer A) and 1.5 M NaBr/100 mM ammonium acetate (Buffer B) in 3:7 acetonitrile: water. Final desalting was performed on a C18 reverse-phase column before drying in a vacuum concentrator. Passenger strands were synthesized similarly on pre-loaded GalNAc support (154 μmol/g). After 5′-dimethoxytrityl removal, oligonucleotides were cleaved with 9:1 NH_4_OH-EtOH for 36 h at room temperature. Purification followed the same SAX and desalting steps as the guide strands.

The 40 μmol scale synthesis of the guide and passenger strands was performed on an AKTA OligoPilot 10, following a reported procedure [35], using NittoPhaseHL UnyLinker solid support (loaded at 317 μmol/g) for the guide strand and pre-loaded GalNAc support (154 μmol/g) for the passenger strand, respectively. The guide and passenger strands were then cleaved, deprotected, and purified using the procedure described above.

Stepwise coupling efficiency for all modifications exceeded 98%, and base-mediated cleavage of phosphonate-containing siRNAs did not exceed 5–6% based on LC-MS analysis under the specified deprotection conditions

### Dual-luciferase reporter assay

The psiCHECK2™ reporter vector (Promega) and Dual-Glo® Luciferase Assay System (Promega) were used to compare the effect of RNAi duplexes described herein below, on on-target versus off-target activity.

The on-target reporter vectors for *Ttr* and *ACTN1* contained a single fully complementary site to the guide strand of the respective siRNAs, inserted into the 3′-untranslated region (3′ UTR) of the Renilla luciferase cassette. For *Ttr*, the sequence was 5′-AAAACAGTGTTCTTGCTCTATAA-3′, while for *ACTN1*, it was 5′-ATGTGTGTTTGCTAGCTCACTTA-3′. The off-target reporter plasmids for both genes contained four seed-complementary sites separated by a 19-nucleotide spacer sequence, also inserted into the 3′ UTR of the Renilla cassette. The seed-complementary sequence for *Ttr* was 5′-GCTCTATAA-3′ with a spacer sequence of 5′-TAATATTACATAAATAAAA-3′, and for *ACTN1*, it was 5′-GCTCACTTA-3′ with a spacer sequence of 5′-TAATATTACAAAAATAAAT-3′.

COS-7 cells (ATCC, Manassas, VA) were grown to near confluence before trypsinization. siRNA duplexes and psiCHECK2 plasmids (Promega) that contain either the on-target or off-target sequences were co-transfected by adding siRNA duplexes at specified concentrations together with 5 μL (10 ng) of psiCHECK2 plasmid per well along with 5 μL of Opti-MEM that had been premixed with Lipofectamine 2000 (2 μg/ml) and then incubated at room temperature for 15 min. The mixture was then added to the cells which had been cultured overnight in 100 μL complete media. Forty-eight hours post-transfection, levels of Firefly luciferase (transfection control) and Renilla luciferase (containing either to on-target or off-target sequence) were measured as per manufacturer's protocol. siRNA activity was determined by normalizing the Renilla signal to the Firefly (control) signal within each well. The magnitude of siRNA activity was then assessed relative to cells that had been transfected with psiCHECK2 plasmid in the absence of siRNA (% control). IC_50_ values were calculated using Prism software under a four-parameter non-linear dose response function.

This assay was used to evaluate relative effects of modifications within each experiment rather than for direct quantitative comparison of IC_50_ values across independent runs.

### Cell culture assays in hepatocytes

Primary hepatocytes were isolated from an 8-week-old male BALB/c mouse using portal vein perfusion. The liver was first perfused with Hank’s Balanced Salt Solution (HBSS) followed by Williams’ Medium E supplemented with Liberase (Sigma-Aldrich) according to the manufacturer's instructions. Cells were washed and purified through a 50% Percoll gradient. Purified hepatocytes were counted and diluted to 1.5 × 10^5 cells/mL in room temperature growth medium. Around 100 μL of the cell suspension was added to each well of a collagen I-coated 96-well culture plate (15 000 cells/well). Immediately after plating, 11 μL of 10X oligonucleotide solution in nuclease-free water was added to the appropriate wells. Cells were treated with a 10-point dose-response curve of siRNAs, with a top concentration of 100 nM and 1:10 serial dilutions. The culture plate was then incubated at 37°C in a humidified atmosphere with 5% CO_2_. After 24 h, cells were lysed for RNA isolation and analysis. Mouse *Ttr* mRNA levels were quantified by RT-qPCR using the QuantStudio 7 Flex Real-Time PCR System (Thermo Fisher Scientific). RT-qPCR reactions (5 μL) containing 1 μL of RNA were performed using AgPath-ID One-Step RT-PCR reagents (Thermo Fisher Scientific) and custom *Ttr* primer-probe sets (Integrated DNA Technologies). Percent untreated control (% UTC) values were calculated using the formula: % UTC = ((Sample QuantityTarget/Sample Quantitynormalization signal) / (Average UTC QuantityTarget/UTC Quantitynormalization signal)) × 100. Half-maximal inhibitory concentration (IC_50_) values were determined using the “log(inhibitor) versus normalized response (variable slope)” function in GraphPad Prism software (v10; GraphPad Software, San Diego, CA).

### Cell culture assays in A431 cells

Off-target effects of modified oligonucleotides complementary to *ACTN1* mRNA were evaluated in human A431 cells (ATCC, CRL-1555). A431 cells were seeded at a density of 10 000 cells per well in 96-well plates and transfected with *ACTN1* siRNAs using a 10-point dose-response curve ranging from 5000 to 0.002 nM (5000, 1000, 200, 40, 8, 1.6, 0.32, 0.064, 0.0128, and 0.002 nM). Transfections were performed using Lipofectamine RNAiMAX (ThermoFisher, 13778150) diluted in Opti-MEM (Gibco, 31985070) according to the manufacturer’s reverse transfection protocol. After a 96-h treatment period, cells were lysed for RNA isolation. Total RNA was purified using a glass fiber filter plate (Pall #5072). Human *ACTN1* mRNA levels were quantified by RT-qPCR using the QuantStudio 7 Flex Real-Time PCR System (Thermo Fisher). RT-qPCR reactions (5 μL) containing 1 μL of RNA were performed using AgPath-ID One-Step RT-PCR reagents (Thermo Fisher) and custom primer-probe sets (Integrated DNA Technologies). Percent untreated control (% UTC) values were calculated using the formula: % UTC = ((Sample QuantityTarget/Sample Quantitynormalization signal) / (Average UTC QuantityTarget/UTC Quantitynormalization signal)) * 100. Half-maximal inhibitory concentration (IC_50_) values were determined using the “log(inhibitor) versus normalized response (variable slope)” function in GraphPad Prism software (v10; GraphPad Software, San Diego, CA).

### Animal studies

Animal experiments were conducted in accordance with the American Association for the Accreditation of Laboratory Animal Care guidelines and were approved by the Animal Welfare Committee (Cold Spring Harbor Laboratory’s Institutional Animal Care and Use Committee guidelines). Animals were housed in micro-isolator cages on a constant 12 h light-dark cycle with controlled temperature and humidity and were given access to food and water ad libitum. Five-week-old C57BL/6J mice (Jackson Laboratories) were treated with a single subcutaneous injection of *Ttr* siRNAs. Additionally, four male C57BL/6 mice were administered subcutaneous injections of PBS, serving as the negative control group for comparison. For RNA analysis, 50–100 mg of liver tissue was homogenized with an Omni Tissue Homogenizer (Omni International) in guanidinium thiocyanate with 8% β-mercaptoethanol. Total RNA was isolated using the PureLink Pro 96 Total RNA Purification Kit (Life Technologies, Carlsbad, CA). qRT-PCR was performed using the StepOne Real-Time PCR system and TaqMan primer probe sets with Express One-Step SuperMix qRT-PCR Kit (Life Technologies, Carlsbad, CA). Primers and probes for the PCR reactions were obtained from Integrated DNA Technologies (IDT). The sequences for the primers and probe used for mouse *Ttr* are forward sequence 5′-CGTACTGGAAGACACTTGGCATT-3′, reverse sequence 5′-GAGTCGTTGGCTGTGAAAACC-3′ and probe sequence 5′- CCCGTTCCATGAATTCGCGGATG-3′. Target RNA levels were normalized to the levels of total RNA measured by RIBOGREEN® RNA Quantitation Reagent (Molecular Probes). Results are presented as percent mouse *Ttr* RNA relative to the amount of *Ttr* RNA in PBS-treated animals (% control). The half maximal effective dose (ED_50_) of each siRNA duplex was calculated using GraphPad Prism 10 software (GraphPad Software, San Diego, CA).

For siRNA tolerability evaluation, single subcutaneous injections of *Ttr* and *Marc1* siRNAs were administered to 7–8 week-old BALB/c mice (Charles River Laboratories). Mice were euthanized 7 days (*Marc1* siRNA) or 14 days (*Ttr* siRNA) after dosing for histopathology assessment. Cardiac blood was collected for clinical chemistry evaluation. *Marc1* primer probe sets: forward sequence 5′- GAAACGGGTGATGGCTTGTA-3′, reverse sequence 5′- GCGGTAGCTCTTCAGTGTTT-3′ and probe sequence 5′- CTTCCTGTCCGAGATGCCAGTGTC-3′.

### RNA sample processing, DGE, and CDF analysis

The RNA samples from both *in vitro* and *in vivo* studies were subjected to differential gene expression (DGE) analysis for 3′-end transcriptome profiling using the QuantSeq 3′ mRNA-Seq Library Prep Kit FWD for Illumina on the Illumina sequencing platform. DGE analysis generated more than 2.5 million unique, mapped reads per sample, providing expression data for over 10 000 unigenes. By comparing control and RNAi duplex-treated groups, we identified unique differentially expressed genes based on criteria of greater than 2-fold change (up- or down-regulation), *P*-value < 0.01, and q-value < 0.1. To effectively visualize these differentially expressed genes, volcano plots were employed, where the *x*-axis represents log2 fold change and the *y*-axis displays -log10 *P*-values.

Cumulative distribution function (CDF) curves of log2 fold change for genes with or without a seed match to each siRNA were used to assess the effect of backbone modifications on siRNA seed match off-target profiles. A left shift of seed match genes relative to the no-seed match baseline on the CDF curves indicates a larger proportion of seed match genes being downregulated compared to background. Estimates of delta (Δ) log2 fold change for seed-match categories were derived from beta coefficients of a linear model regressing log2 fold change against each seed match type. Statistical significance was evaluated using two-tailed t-statistics. Seed-match genes were identified using TargetRank (http://hollywood.mit.edu/targetrank/).

### Histology

Livers were fixed in 10% Neutral Buffered Formalin and processed using a mouse tissue protocol on a Sakura Tissue Tek tissue processor. After embedding, 4-micron-thick sections were cut, air-dried overnight, and further dried at 60°C for 1 h. The sections were stained using a Leica Spectra stainer with Gills II Hematoxylin (Leica/Surgipath cat# 3801520) for 4 min, followed by differentiation in 0.5% Acid Alcohol for 1 second. Counterstaining was performed with Eosin (Leica/Surgipath cat# 3801600) for 2 min. Stained slides were scanned at 20X resolution using a Hamamatsu S360 scanner and analyzed by a board-certified pathologist.

### Melting temperature (*T*_m_) analysis

Melting temperature measurements were conducted using siRNA duplexes prepared at a concentration of 1 μM in 0.1X PBS buffer. The duplexes were initially denatured at 95°C and then slowly cooled to the starting temperature of the experiment, which was set at 15°C. The thermal denaturation temperatures were measured in quartz cuvettes with a pathlength of 1.0 cm on a Cary 100 UV/visible spectrophotometer equipped with a Peltier temperature controller. Absorbance was recorded at 260 nm as a function of temperature, utilizing a temperature ramp of 0.5°C/min. T_m_ values were determined using the hyperchromicity method incorporated into the instrument's software, which analyzes the increase in absorbance that occurs when double-stranded nucleic acids separate into single strands upon heating.

### Molecular dynamics simulations

The human Argonaute 2 structure was obtained from the 5T7B PDB entry. Missing loops and modified oligonucleotides were added using the Discovery Studio Simulation Client v23.2.500. To construct the guide strand, each modified oligonucleotide was built using Gauss View 6.0; the structure was optimized, and charges were calculated using the HF/6-31G* level of theory and basis set as implemented in Gaussian 16 C.01. Charge fitting was performed with the antechamber tool from AmberTools 20 using the RESP methodology. Missing parameters for each modified nucleotide were extracted from the General Amber Force Field (GAFF2). A topology and coordinates file was generated using the LEaP program, employing the OL21, OL3, and FF14SB AMBER force fields for DNA, RNA, and protein structure, respectively. NaCl ions were added to neutralize the charge, and additional ions were included to achieve a final concentration of 150 mM. Sufficient Mg^2+^ ions were added to reach a concentration of 20 mM, with ions described by the Joung-Cheatham parameters. The system was embedded in a truncated octahedral periodic cell unit, with water molecules added using the OPC model. The solute-edge buffer of the box was set to 10 Å. Initial minimization and heating of the system were performed using a nine-step protocol as implemented in the AmberMDPrep program. Each simulation was conducted with three independent replicas, each utilizing different seeds and randomized waters for approximately 1 μs, using the PMEMD CUDA implementation found in AMBER 20. Analysis was performed with the GIT version of CPPTRAJ.

## Results

### Synthesis of alkyl phosphonate-modified siRNAs

**Figure 1.**
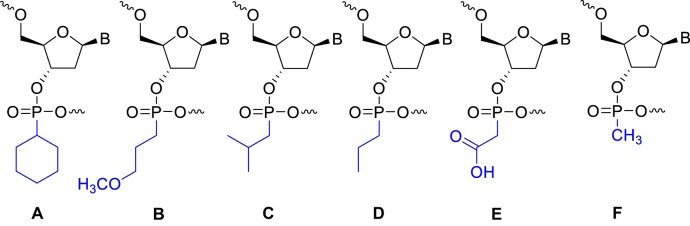
Charge-neutral alkyl phosphonate linkages of different sizes: (**A**) cyclohexyl phosphonate (cHex), (**B**) methoxypropyl phosphonate (MOP), (**C**) isobutyl phosphonate (iBu), (**D**) propyl phosphonate (Propyl), (**E**) phosphonoacetate (PACE), and (**F**) methyl phosphonate (MP).

Non-natural internucleotide linkages have been extensively researched and demonstrated to influence the biophysical and biological properties of therapeutic oligonucleotides [29, 31]. Various chemically modified backbones have been successfully incorporated into antisense oligonucleotides (ASOs) [36, 37] and siRNAs [38] to enhance enzymatic stability or drug-like properties. While a comprehensive review of these diverse backbone modifications is beyond the scope of this manuscript, their pivotal role in advancing oligonucleotide therapeutics cannot be overstated. In previous work, we described the synthesis of charge-neutral alkyl phosphonate linkages and their incorporation into gapmer ASOs (Fig. 1A–D) [29, 33]. Our findings illustrated that site-specific replacement of phosphorothioate with alkyl phosphonate linkages enhances the therapeutic profile of ASOs [33]. We hypothesized that these modifications could similarly impact siRNA therapeutics by inducing backbone perturbations in the guide strand’s seed region, potentially altering Argonaute 2 (Ago2) interactions. This could modify mRNA binding, reducing off-target activity.

For the initial screens, we selected a previously reported mouse transthyretin (*Ttr*)-targeting siRNA sequence as the tool, which has a validated off-target profile (Table 1, line 1) [27]. Although modifications can be incorporated at any position along the guide strand, previous data have shown that those modulating seed affinity are most effective at positions 5, 6, or 7 from the 5′-end [20, 21, 27]. Consequently, we limited the initial screens to internucleotide linkages at positions 5–6 and 6–7 (Table 1, line 2 and 3).

**Table 1.** siRNA sequences and chemistry. Red: 2**′-**OMe, Green: 2**′-**F, Black: 2**′**-Deoxy, Orange: 2**′**-*O*-MOE, GalNAc: N*-*Acetylgalactosamine, VP: 5**′**-(E)-vinylphosphonate. All chemical linkages are phosphodiester unless specifically marked as “s” (phosphorothioate) or “x” (alkyl phosphonate)

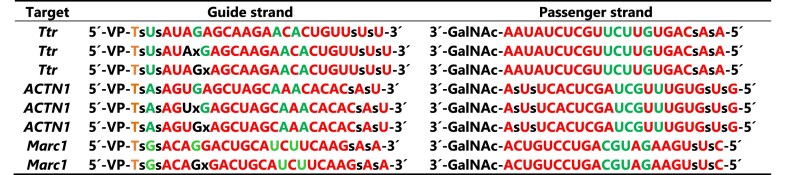

**Scheme 1.**
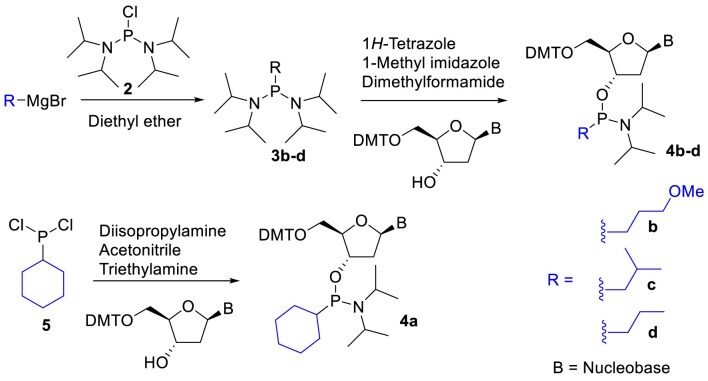
Synthesis of nucleoside phosphonamidites **4a-d**. DMT: 4,4**′**-Dimethoxytrityl, B: -Isobutyrylguanine or N^6^-Benzoyladenine.

The alkyl phosphonate modified nucleoside analogues **1a-d** (Fig. 1) were synthesized following a previously described procedure and incorporated into the guide strand utilizing the corresponding phosphonamidites [29, 33]. In brief, commercially available or synthesized alkyl Grignard reagents were initially reacted with bis(diisopropylamino)chlorophosphine **2** to yield alkyl-bis(diisopropylamino)phosphine **3b-d** (Scheme 1). These reagents were then isolated through extraction into hexanes and subsequently reacted with N (amino)-protected 2**′**-deoxy-5**′**-dimethoxytrityl-adenosine or guanosine to yield the desired nucleoside phosphonamidites **4b-d** (Scheme 1). Due to solubility issues, nucleoside phosphonamidite **4a** couldn’t be obtained from reagent **2**. Instead, it was synthesized by reacting commercially available cyclohexyldichlorophosphine **5** (Scheme 1) with the corresponding nucleoside in the presence of diisopropylamine to yield phosphonamidite **4a**. These phosphonamidites were subsequently introduced into oligonucleotides using standard phosphoramidite protocols, followed by deprotection with 10% diethylamine in 9:1 ammonium hydroxide: ethanol for 36 h at room temperature (Supplementary Table S1).

### Alkyl phosphonate at position 6-7 enhances the specificity of transthyretin siRNAs

**Figure 2.**
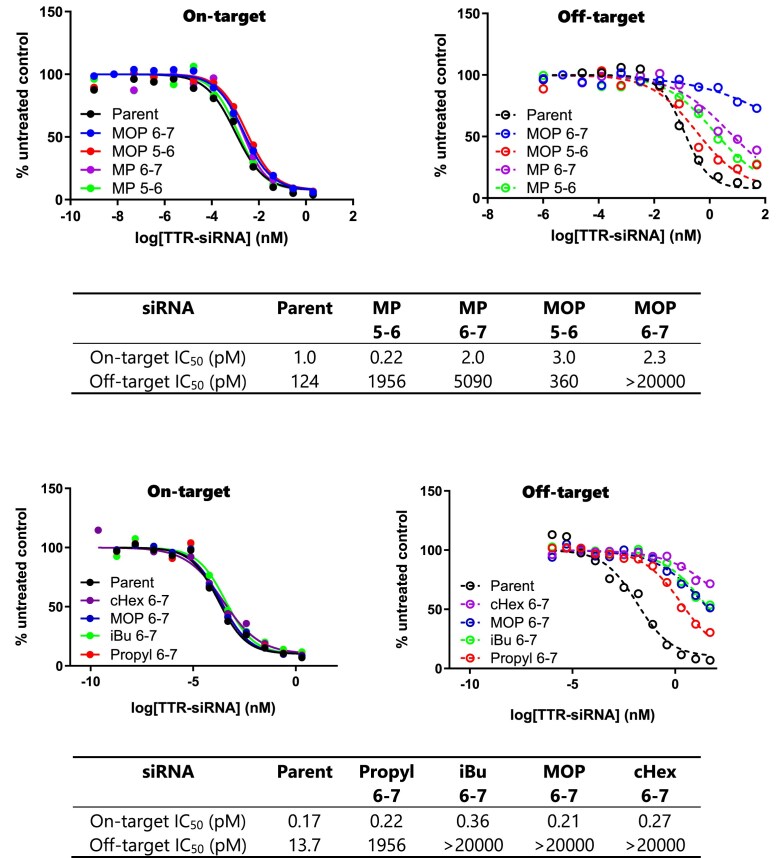
Dual-luciferase reporter assay results for *Ttr* siRNAs modified with Top: MP and MOP at positions 5–6 and 6–7; Bottom: propyl, iBu, MOP, and cHex at positions 6–7. Solid line: on-target; dashed line: off-target.

To assess off-target effects, we relied on the previously reported Dual-Luciferase Reporter Assay System (Promega) [20, 39], which provides a normalized value for on-target and off-target activities based on the ratio of Renilla luminescence to Firefly luminescence (see the “Material and Methods” section for details). We began our screening process by comparing a small methylphosphonate (MP, Fig. 1F) with a relatively bulkier MOP (Fig. 1B). When positioned at internucleotide linkages 5–6 and 6–7 of the *Ttr* guide strand, both modifications influenced off-target activity at both positions. However, the impact was notably stronger at the internucleotide linkages of 6–7. Furthermore, the bulkier modification (MOP) showed a more significant ability to mitigate off-target effects (Fig. 2). Building on these findings, we designed a subsequent experiment in which alkyl phosphonates bearing 3- (Propyl, Fig. 1D), 4- (iBu, Fig. 1C), 5- (MOP, Fig. 1B), and 6- (cHex, Fig. 1A) atoms were synthesized and placed at positions 6–7. Effective off-target mitigation was observed for all these modifications, with the effects being most pronounced for the bulkier modifications. It is noteworthy to mention that all tested siRNAs remained active, as determined by the dual luciferase assay, and none of them lost on-target activity due to backbone modification (Fig. 2). Despite variability in parent IC_50_ values across experiments in the Dual-Luciferase Assay, modified siRNAs consistently showed improved off-target profiles relative to the parent within each run. To assess the effect of the charge, we also prepared a phosphonoacetate [40] (Fig. 1E, PACE)-modified guide strand using commercially available phosphonamidite reagents and evaluated it in a similar manner. PACE provided no benefit and performed worse than propyl, suggesting charge is not a key factor. (Supplementary Fig. S1).

**Figure 3.**
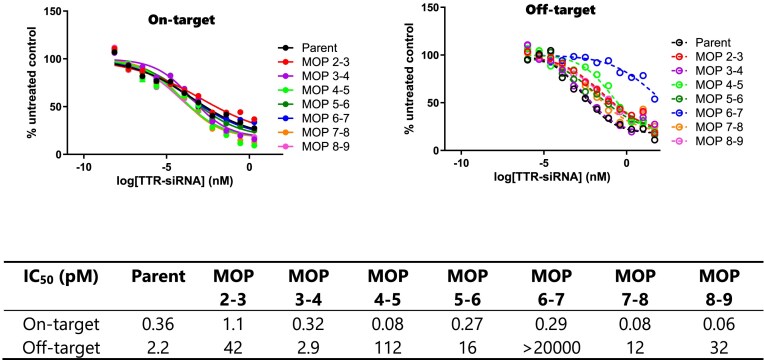
Dual-luciferase reporter assay results for *Ttr* siRNAs modified with a single MOP at positions 2–9 of the guide strand. Solid line: on-target; dashed line: off-target.

To further confirm positions 6–7 as the optimal site, we conducted a full seed walk, incorporating a single MOP at positions 2–9 of the siRNA guide strand. Subsequent evaluation through dual luciferase assay revealed that none of the tested positions surpassed the efficacy observed at positions 6–7, reinforcing our initial finding (Fig. 3).

**Figure 4.**
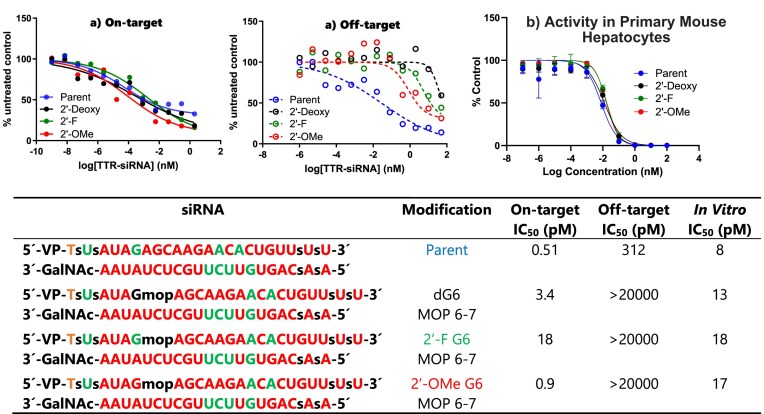
(**A**) Dual-luciferase reporter assay results for *Ttr* siRNAs modified with 2**′-**OMeG, 2**′-**FG, dG at position 6 and MOP at 6–7. Solid line: on-target; dashed line: off-target. (**B**) *In vitro* activities of the same siRNAs in primary mouse hepatocytes (free uptake, 24 h).

Considering that the nucleoside phosphonamidites used in our screening assays exclusively consisted of 2**′**-deoxy, we wondered whether the observed screening outcomes would be relevant to the common siRNAs, which often contain 2**′**-*O*-methyl (2**′-**OMe) and 2**′**-fluoro (2**′**-F) ribose substitutions. To address this, we synthesized guanosine phosphonamidites containing 2′-OMe and 2′-F, enabling a comparative assessment of their on-target and off-target activities. Using the same *Ttr* siRNA and luciferase assay as before, we conducted a direct comparison experiment, analyzing their on-target and off-target activities side by side. As shown in Fig. 4A, all three nucleosides effectively mitigated off-target activity in combination with MOP. Additionally, all three siRNAs exhibited activity in the dual luciferase assay. It is noteworthy that while this assay proved invaluable for modification screening, it lacked the precision necessary for accurate IC_50_ determination. To compare on-target activities, we assessed all three siRNAs in a dose-response assay in mouse primary hepatocytes, revealing nearly equal potencies *in vitro* (Fig. 4B). Therefore, we continued with 2′-deoxynucleosides for the rest of the experiments.

**Figure 5.**
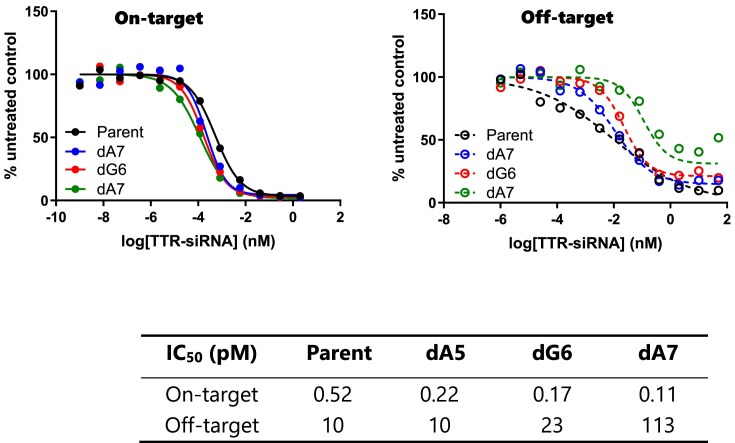
Dual-luciferase reporter assay results for *Ttr* siRNAs modified with dA or dG at positions 5–7 of the guide strand. Solid line: on-target; dashed line: off-target.

Continuing our investigation, we aimed to determine the viability of employing a single 2′-deoxy nucleoside for off-target mitigation. To address this, we synthesized *Ttr* siRNAs containing 2′-deoxy nucleosides at positions 5–7 of the guide strand and assessed their on-target and off-target activities in the absence of alkyl phosphonates. As depicted in Fig. 5, a single 2′-deoxy nucleoside alone was not efficient. While we observed some improvements at position 7, overall, the effects were not comparable to those observed when alkyl phosphonate was present. Thus, we demonstrated that alkyl phosphonate plays an indispensable role in improving the specificity of siRNA.

### Alkyl phosphonate at positions 6–7 enhances the specificity of alpha-actinin 1 siRNAs

**Figure 6.**
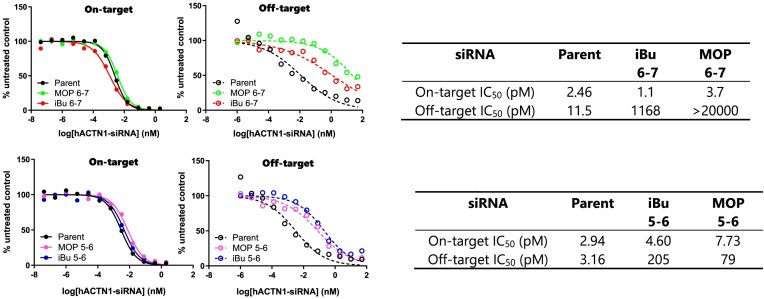
Dual-luciferase reporter assay results for *ACTN1* siRNAs modified with Top: iBu and MOP at position 6-7; Bottom: iBu and MOP at positions 5–6. Solid line: on-target; dashed line: off-target.

While alkyl phosphonates effectively improved the specificity of *Ttr* siRNA, it was essential to validate these findings with a different siRNA sequence. During our routine screening, we identified a promiscuous human siRNA targeting alpha-actinin 1 (*ACTN1*) (refer to Table 1, line 4). Subsequently, we functionalized it with MOP (depicted in Fig. 1B) and iBu phosphonate (shown in Fig. 1C) at positions 5–6 and 6–7, respectively, as outlined in Table 1, lines 5 and 6, and subjected them to dual luciferase analysis. Much like the *Ttr* siRNA, both modifications were effective in mitigating off-target effects, with optimal efficiency observed when placed at the internucleotide linkage 6–7 (Fig. 6). We also validated our results using a third siRNA sequence, targeting mouse hydroxyacid oxidase 1 (*Hao1*) [27], as depicted in Supplementary Fig. S2. Taken together, these results suggest that alkyl phosphonates are compatible with multiple siRNAs, further expanding their applicability in reducing off-target effects.

### Alkyl phosphonate-modified transthyretin siRNAs retain activity *in vitro* and *in vivo* while enhancing specificity

**Figure 7.**
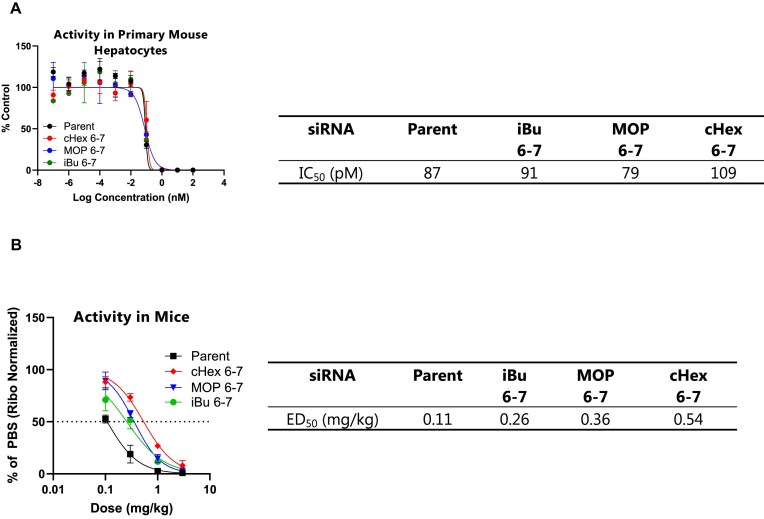
(**A**) *In vitro* activities of the *Ttr* siRNAs modified with iBu, MOP and cHex at positions 6–7 in primary mouse hepatocytes (free uptake, 24 h), (**B**) *In vivo* activities of the *Ttr* siRNAs modified with iBu, MOP, and cHex at positions 6–7 in mice,

With the completion of the dual luciferase assay screenings, we identified three alkyl phosphonates, iBu, MOP, and cHex that demonstrated the most promising off-target mitigation potentials for further analysis. First, we confirmed the *in vitro* activity of *Ttr* siRNAs containing these modifications at positions 6–7 (as outlined in Table 1, line 3) in mouse primary hepatocytes. As seen in Fig. 7A, all three siRNAs maintained their efficacy *in vitro*, with IC_50_ values comparable to the parent compound.

Next, we focused on verifying the *in vivo* activity to refine our selection. To achieve this, we administered a single subcutaneous injection of *Ttr* siRNAs containing iBu, MOP, and cHex at positions 6–7 to mice at doses of 0.1, 0.3, 1, and 3 mg/kg once and euthanized the animals after one week. *Ttr* mRNA expression was then quantified by qPCR. As illustrated in Fig. 7B, all three siRNAs maintained their *in vivo* activity, with iBu exhibiting the most favorable ED_50_, followed by MOP and then cHex. Consequently, we chose iBu and MOP for further experimentation. Given the nature of alkyl phosphonates, some reduction in on-target activity was anticipated due to changes in the backbone structure. Nevertheless, we were pleased to observe that ED_50_ values of these siRNAs were comparable to that of the parent siRNA.

**Figure 8.**
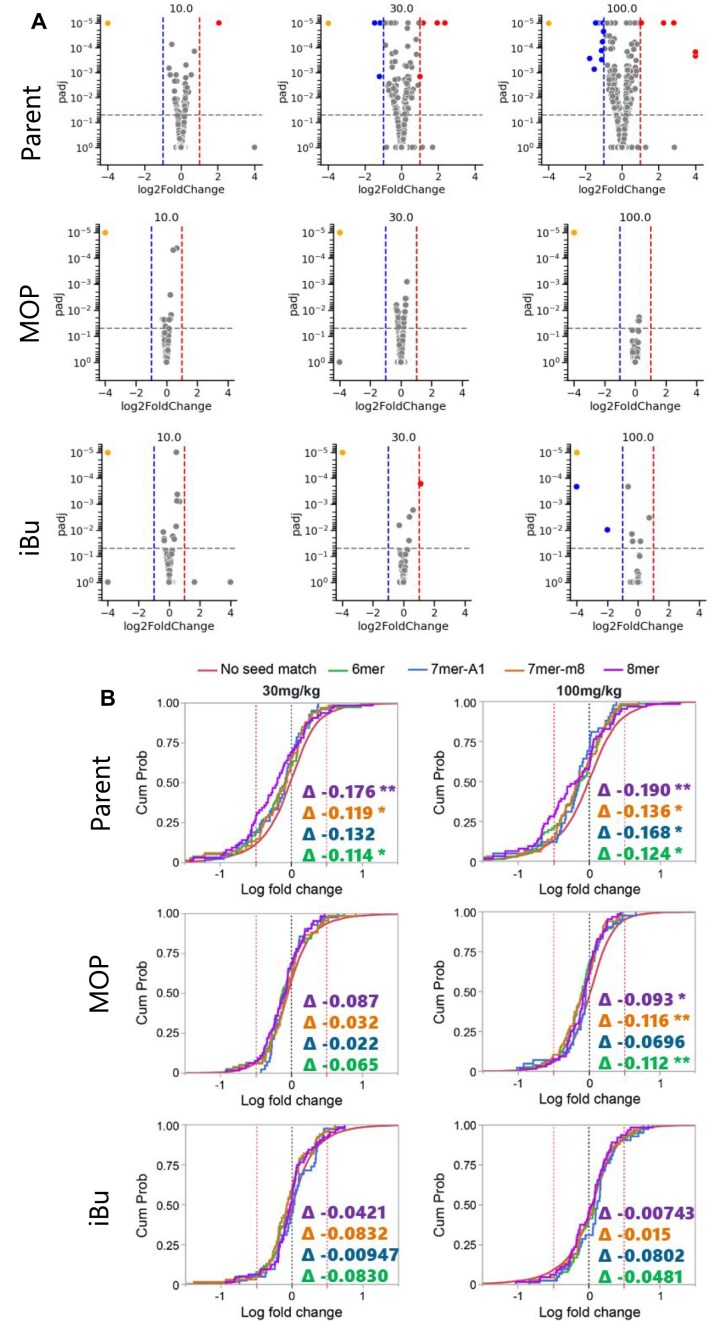
(**A**) Volcano plots of DEGs in mouse liver after *Ttr* siRNA treatment (10, 30, and 100 mg/kg, 7 days). Top: Parent, Middle: MOP, Bottom: iBu. Orange: On-target (*Ttr*), Red: Induced DEGs, Blue: Depleted DEGs, Grey: Not significant. (**B**) Expression comparison for transcripts with 8mer or 6/7mer seed matches vs. no match after *Ttr* siRNA (30 and 100 mg/kg, 7 days). Delta values: CDF shifts (negative = downregulation). Significance: *P < 0.05, **P < 0.01, ***P < 0.001; no stars = *P* ≥ 0.05.

To evaluate off-target mitigation *in vivo*, we administered escalating doses of iBu and MOP-modified *Ttr* siRNAs (0.1, 0.3, 1, 3, 10, 30, and 100 mg/kg) to mice. After 1 week, the animals were euthanized, and liver tissue was extracted for DGE analysis, following the procedures outlined in the “Materials and Methods” section. Genes were identified as differentially expressed (DEG) if they met the criteria of a fold change greater than 2-fold down or upregulation, a *P*-value less than 0.01, and a q-value less than 0.1. This identification was made through a comparison of the saline and the siRNA treated groups. The DEGs were then depicted in volcano plots, with red indicating upregulation, blue indicating downregulation, and grey indicating no significant change in expression. The volcano plots illustrating the top three doses are displayed in Fig. 8A. While the parent *Ttr* siRNA exhibited a dose-dependent increase in the number of DEGs, both iBu and MOP modifications effectively mitigated off-target effects. We observed a dose-dependent decrease in the number of DEGs and also a reduction in the number of subthreshold gene expressions. These results strongly support the notion that these modifications can exert their functional influence *in vivo*.

To further investigate the efficacy of alkyl phosphonates in reducing miRNA-like effects, we examined the expression of transcripts with 8mer or 6/7mer seed matches [41] (identified using TargetRank, see the “Materials and Methods” section) to *Ttr* siRNAs at their highest doses (30 and 100 mg/kg). These transcripts were then compared to those with no seed matches, serving as the baseline, using a CDF curve. As shown in Fig. 8B, the parent *Ttr* siRNA shows a left shift of the 8mer and 6/7mer seed match genes over the baseline on the CDF curve, indicating a larger proportion of seed-match genes being downregulated. However, this shift is less pronounced for the modified siRNAs. Collectively, these observations demonstrate the efficacy of alkyl phosphonates in substantially reducing miRNA-like effects.

### Alkyl phosphonate-modified alpha-actinin 1 siRNAs retain activity and improve specificity *in vitro*

**Figure 9.**
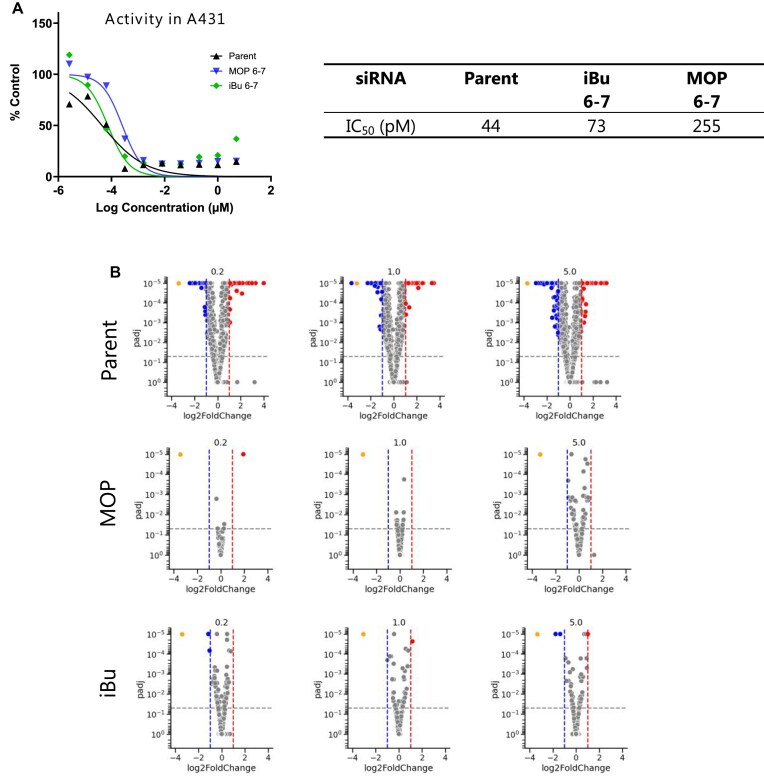
(**A**) *In vitro* activities of the *ACTN1* siRNAs modified with iBu and MOP at positions 6–7 in A431 cell line (lipid transfection, 96 h), (**B**) Volcano plots of DEGs following *ACTN1* siRNA treatment at 0.2, 1, and 5 μM doses. Top: Parent, Middle: MOP, Bottom: iBu. Orange: On-target (*ACTN1*), Red: Induced DEGs, Blue: Depleted DEGs, Grey: Not significantly changed.

Next, we evaluated the in vitro activity and off-target mitigation potential of *ACTN1* siRNAs modified with iBu and MOP at positions 6-7 (Table 1, line 6) in the A431 epidermoid carcinoma cell line. Consistent with our findings for *Ttr* siRNAs, iBu demonstrated better on-target activity compared to the MOP, with an IC_50_ closer to that of the parent siRNA (Fig. 9A).

To assess transcriptome changes induced by parent and modified *ACTN1* siRNAs, we performed DGE analysis as described in the “Materials and Methods” section. We observed a dose-dependent increase in the number of differentially expressed genes (DEGs) for the parent *ACTN1* siRNA. In contrast, both modified siRNAs effectively mitigated this effect, reducing the number of DEGs across the dose range, with the reduction becoming more pronounced at higher doses (Fig. 9B). Interestingly, while we attempted to generate CDF plots for the *ACTN1* siRNAs, the left shift trend observed *in vivo* was not notably discernible *in vitro*. This discrepancy may be attributed to the complex liver microenvironment and the GalNAc conjugated siRNA’s propensity for hepatic accumulation, which could play a crucial role in manifesting the observed effects *in vivo*.

### Alkyl phosphonate at positions 6–7 enhances the therapeutic profile and reduces hepatotoxicity of *Ttr* and *Marc1* siRNAs

Toxicity remains a significant concern in the development of oligonucleotide therapeutics, including both ASOs and siRNAs. Similar to high affinity liver targeting ASOs, where hybridization-based off-target effects can lead to liver toxicity [42, 43], siRNAs have also been shown to induce hepatotoxicity through their off-target interactions [25, 27]. Previous studies have provided crucial insights into the mechanisms of siRNA-induced hepatotoxicity. Notably, seed region swapping experiments between toxic and non-toxic siRNAs have demonstrated that the seed region plays a pivotal role in hepatotoxicity, with minimal contribution from the overall siRNA chemistry [25]. Furthermore, chemical modifications in the seed region of siRNAs have shown promise in reducing hepatotoxicity. For instance, rat studies have revealed that siRNAs modified with (S)-GNA in the seed region exhibited significantly decreased or even complete attenuation of liver enzyme elevations, including alanine aminotransferase (ALT), aspartate aminotransferase (AST), and glutamate dehydrogenase (GLDH), compared to their unmodified counterparts [27]. Inspired by these promising results, we sought to investigate whether our novel alkyl phosphonate modifications could similarly impact hepatotoxicity.

**Figure 10.**
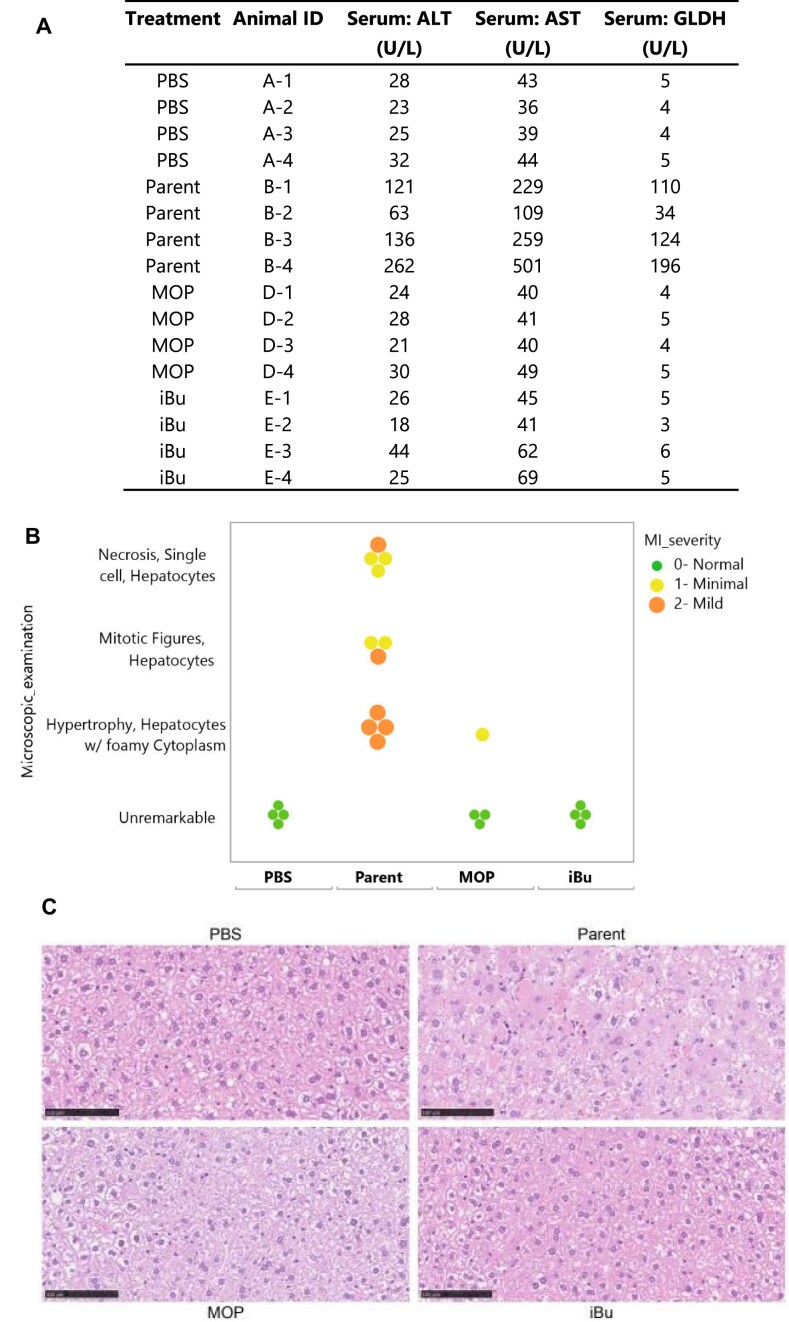
Toxicity assessment of parent and modified *Ttr* siRNAs in mice. Mice received a single 100 mg/kg subcutaneous dose of parent, MOP-modified, or iBu-modified *Ttr* siRNAs. (**A**) Serum levels showed mild increases with the parent compound, which correlated with (**B** and **C**) hepatocyte necrosis, increased mitotic figures, and hypertrophy with foamy cytoplasm. Modified siRNAs showed no enzyme elevations or histopathological changes.

To conduct the toxicity studies, we used two *Ttr* siRNAs, iBu and MOP, at positions 6–7, previously tested in the DGE experiment, along with the parent siRNA as a control. Mice (4 per group) were dosed subcutaneously at 100 mg/kg once and euthanized after two weeks. Organs were harvested, and blood samples were collected from all four animals for analysis. The results showed that the parent *Ttr* siRNA at 100 mg/kg was not well tolerated, as all animals exhibited mild necrosis and regeneration of hepatocytes which correlated with mild increases in AST/ALT and mild-to-moderate increases in GLDH (Fig. 10A). Additionally, all animals presented with mild hypertrophy and foamy cytoplasm (Fig. 10B and C). In contrast, treatment with the *Ttr* siRNA modified with MOP at 100 mg/kg was well tolerated. Only one animal presented with mild hypertrophy and foamy cytoplasm and increased hematoxylin staining, without correlated changes in clinical chemistries (Fig. 10B and C). Similarly, the *Ttr* siRNA modified with iBu at 100 mg/kg was also well tolerated, with no histopathological findings (Fig. 10B and C). Overall, our findings confirmed that the parent *Ttr* siRNA, previously reported to be toxic after repeat dosing in rats, is also toxic in mice, validating our study design. More importantly, we demonstrated that alkyl phosphonate modifications can effectively attenuate hepatotoxicity, as evidenced by reduced liver enzyme elevations and histopathological changes (Fig. 10A–C).

**Figure 11.**
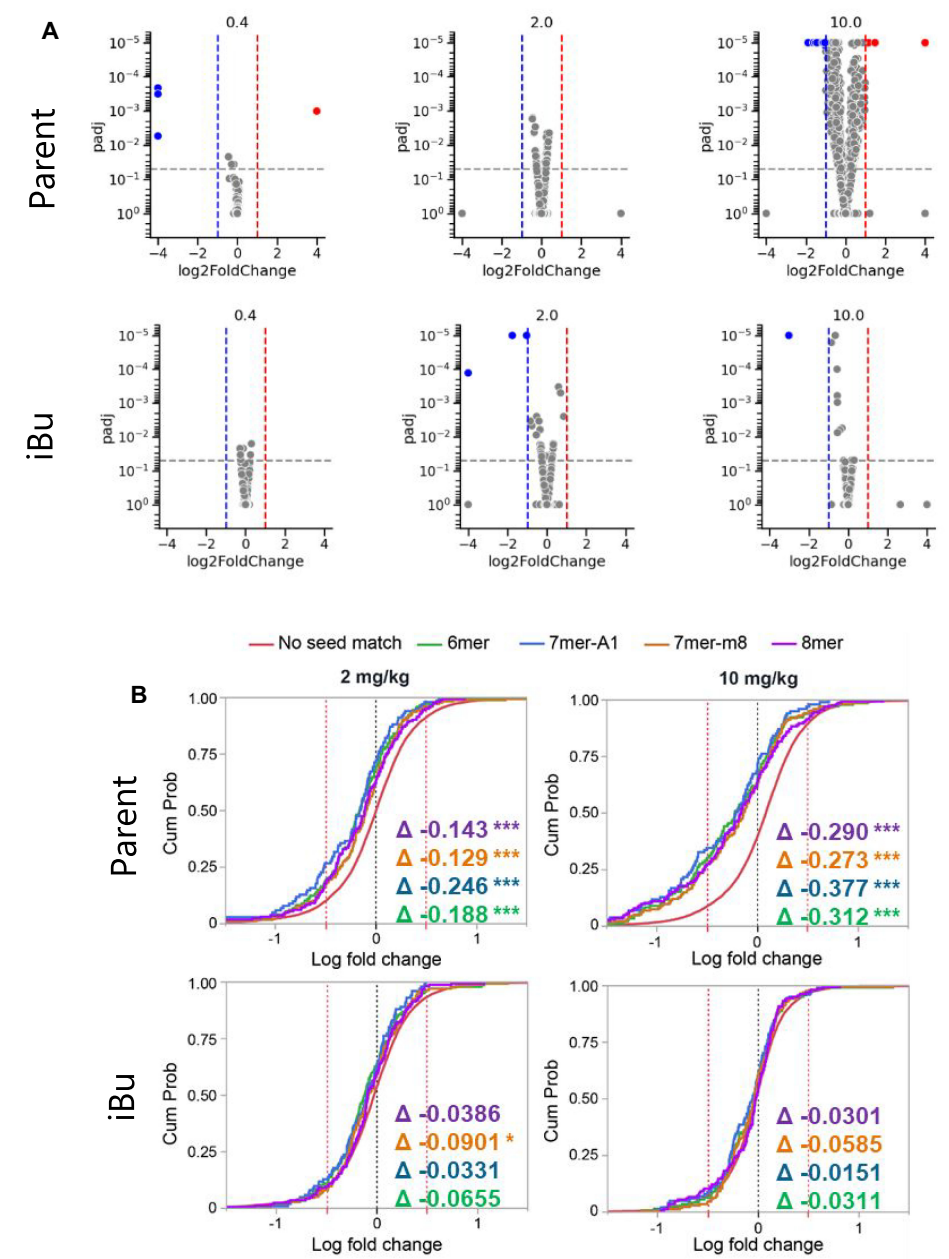
(**A**) Volcano plots of DEGs in mouse liver after *Marc1* siRNA treatment (0.4, 2, and 10 mg/kg, 3 days). Top: Parent, Bottom: iBu. Red: Induced, Blue: Depleted, Grey: Not significant. (**B**) Expression comparison for transcripts with 8mer or 6/7mer seed matches versus no match after *Marc1* siRNA (2 and 10 mg/kg, 3 days). Delta values: CDF shifts (negative = downregulation). Significance: *P < 0.05, **P < 0.01, ***P < 0.001; no stars = *P* ≥ 0.05.

**Figure 12.**
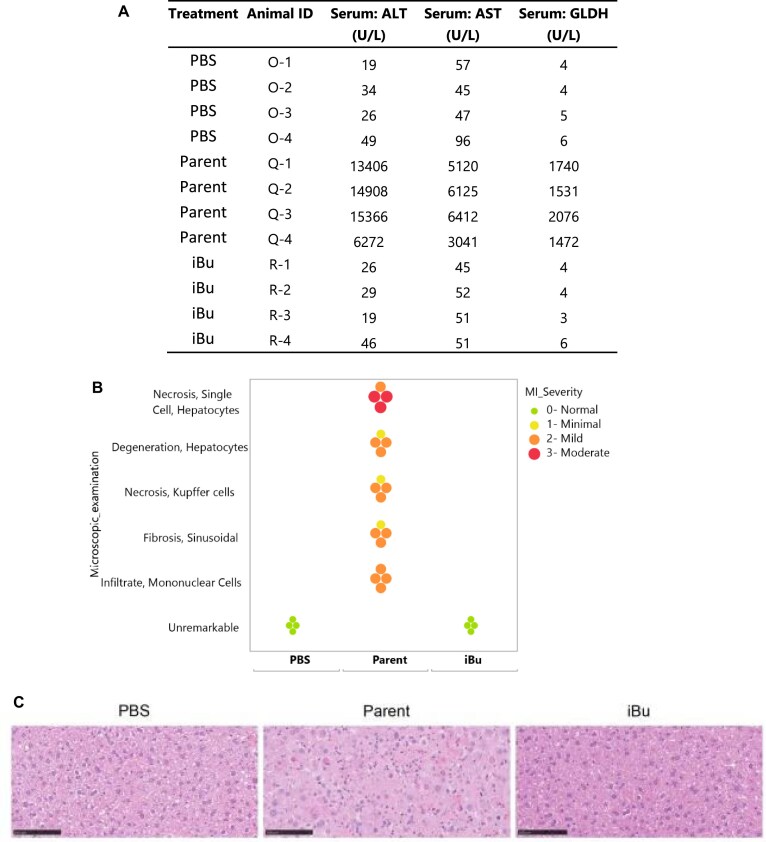
Toxicity assessment of parent and iBu-modified *Marc1* siRNAs in mice. A single 10 mg/kg subcutaneous dose was administered, and serum liver enzymes and histopathology were evaluated after 1 week. (**A**) The parent caused significant liver enzyme increases, which correlated with (**B**), **C**) moderately increased hepatocyte single-cell necrosis, degeneration, and proinflammatory changes (mononuclear infiltrates, sinusoidal fibrosis). The iBu-modified siRNA showed no enzyme elevations or histopathological changes.

To further validate these results, we sought to test a second sequence. In our routine screening, we identified a mouse targeting mitochondrial amidoxime reducing component 1 (*Marc1*) siRNA (Table 1, line 7) whose toxicity was manifested at lower doses (∼1 mg/kg) and at an earlier time point (4 days) compared to the *Ttr* siRNA. To confirm that this toxic siRNA indeed has an off-target profile, we modified it with iBu at positions 6–7 (Table 1, line 8) and dosed it at 0.08, 0.4, 2, and 10 mg/kg alongside the parent siRNA. We intentionally kept the treatment time short to avoid any influence from the early onset of toxicity. After 3 days, the animals were euthanized, and liver tissue was extracted for *in vivo* activity and DGE analysis, following the procedures outlined in the “Materials and Methods” section. In terms of activity, the iBu-modified *Marc1* siRNA demonstrated *in vivo* activity comparable to the parent siRNA, reaffirming that the introduction of iBu at position 6-7 of the guide strand is well tolerated (Supplementary Fig. S3). The DGE analysis for the same study was then visualized in volcano plots. As shown in Fig. 11A, while the parent *Marc1* siRNA exhibited a dose-dependent increase in the number of DEGs, iBu effectively countered the observed off-target effects, leading to a notable reduction in both DEGs and subthreshold gene expressions. Additionally, the CDF curve plotted demonstrated a larger proportion of seed-match genes being downregulated, which was again consistent with miRNA-like off-target effects (Fig. 11B). In a follow-up study, mice were administered 10 mg/kg of the parent *Marc1* siRNA for one week, which resulted in mild-to-moderate liver toxicity, as indicated by significant elevations in liver enzyme levels (Fig. 12A). All animals in this group exhibited signs of liver damage, including moderate hepatocyte single-cell necrosis, degeneration, and pro-inflammatory changes, such as mononuclear cell infiltrates and sinusoidal fibrosis (Fig. 12B and C). In contrast, treatment with the iBu-modified siRNA led to a substantial reduction in enzyme levels, with ALT and AST decreasing by over 99%, returning to normal physiological ranges (Fig. 12A). Mice treated with the iBu-modified siRNA showed no histopathological abnormalities in the liver (Fig. 12B and C). These results demonstrate that backbone modifications can effectively reduce siRNA-induced hepatotoxicity while maintaining efficacy, potentially leading to safer RNA-based therapeutics.

### Melting temperature studies suggest additional factors influencing modified siRNA behavior

To elucidate the mechanism of action and explain the observed properties of alkyl phosphonates of varying sizes, we conducted melting temperature (T_m_) experiments on both the parent and modified *Ttr* siRNAs. The results of these experiments are summarized in Table 2. Our analysis revealed that the introduction of phosphonate modifications in the seed region generally led to a decrease in *T*_m_ values compared to the unmodified parent siRNA. However, the observed changes in *T*_m_ did not consistently predict the extent of off-target mitigation or on-target efficacy. Interestingly, siRNAs with similar *T*_m_ values demonstrated markedly different on-target profiles. For instance, the change in *T*_m_ (ΔT_m_) observed when placing a methylphosphonate (MPO) modification at positions 5–6 and 6–7 was nearly identical. However, the 6–7 position proved more effective in mitigating off-target effects. This suggests that factors beyond thermal stability play a crucial role in determining siRNA specificity and efficacy. Furthermore, the ΔT_m_ values observed for different alkyl chains such as cyclohexyl (cHex), methoxypropyl (MOP), isobutyl (iBu), and propyl (Prp) phosphonates were within half a degree of each other. Despite these similar *T*_m_ values, these modifications exhibited varying degrees of off-target mitigation and on-target potency. While *T*_m_ is a quantifiable parameter that has been previously used to explain siRNA behavior, our findings suggest that it alone is insufficient to fully elucidate the complex mechanisms at play. We propose that additional factors, such as conformational changes and interactions with the Argonaute 2 (Ago2) protein, significantly influence siRNA performance. These subtle yet crucial aspects are not easily measurable through standard *T*_m_ experiments. Therefore, while T_m_ analysis provides valuable insights, it should be considered in conjunction with other factors to comprehensively understand the behavior of modified siRNAs. In conclusion, our *T*_m_ experiments highlight the complex relationship between thermal stability and siRNA functionality. The results underscore the need for a multifaceted approach in designing and optimizing siRNA modifications, taking into account not only *T*_m_ but also other molecular interactions and conformational changes that may impact siRNA efficacy and specificity.

**Table 2.** The table presents melting temperature differences for modified *Ttr* siRNAs compared to the parent. Each siRNA includes a backbone modification at designated positions, along with a deoxy sugar modification downstream, as previously described

Modification	Position	Δ*T*_m_ (°C)
MPO	5–6	­3.63
MPO	6–7	­3.55
MPO	7–8	­3.20
Prp	6–7	­4.86
iBu	6–7	­4.69
MOP	6–7	­4.31
cHex	6–7	­5.11

### Molecular dynamics simulations reveal modified backbone effects on hAgo2-RNA dynamics

**Figure 13.**
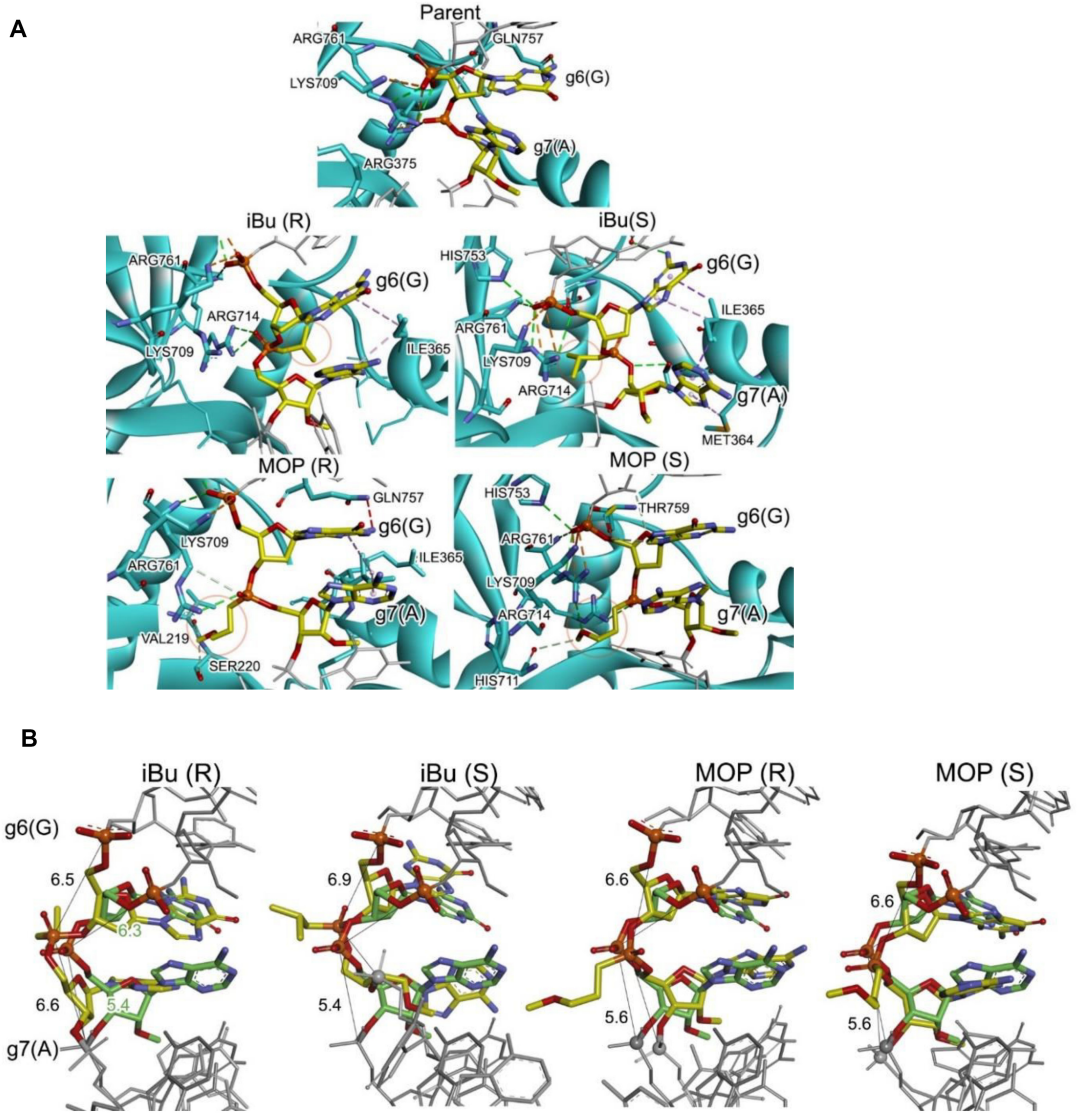
MD simulations reveal conformational changes in hAgo2-guide RNA interactions upon backbone modification. (**A**) Representative structure from the most populated cluster. hAgo2 protein is shown in cyan, with guide RNA carbon atoms in yellow. The backbone modification site is highlighted by a red circle. Key residues (Arg375, Lys709, Arg714, and Arg761) and their interactions with the RNA backbone are emphasized. (**B**) Structural comparison of parent (green) and modified (yellow) guide strands in hAgo2. Overlay of representative structures from cluster analysis, aligned using the parent strand backbone. Phosphate-phosphate distances (Å) at positions 6–7 and 7–8 are indicated, highlighting conformational changes induced by backbone modifications.

Molecular dynamics (MD) simulations and X-ray crystallography provide complementary insights into the complex interactions between human Argonaute 2 (hAgo2) and guide RNA, particularly focusing on the seed region and backbone modifications. MD simulations reveal specific non-covalent interactions between the parent *Ttr* guide and residues of hAgo2, such as Arg375, Lys709, Gln757, and Arg761, primarily involving the phosphate backbone. These interactions align with those observed in crystal structures (PDB 5T7B) [44]. However, when neutral backbone modifications, such as isobutyl (iBu) or methoxypropyl (MOP) phosphonates, are introduced, the guide strand undergoes significant reorientation. Notably, contacts with Lys709 and Arg761, which lock the phosphate at position 5–6, remain conserved, acting as a crucial anchor, while other interactions are altered to accommodate the modified backbone. For example, the phosphate at positions 6–7 of the parent guide interacts with Arg375 but shifts to accommodate Arg714 and Arg761 upon modification (Fig. 13A). Such reorganization is accompanied by structural deviations, with RMS values ranging from 4.0 to 5.7 Å, reflecting the altered dynamics in the seed region. The phosphate-phosphate distances are observed to be similar in all cases with a ∼1.2 Å reduction in the 7–8 position that suggests increased structural deviation due to the modified backbone (Fig. 13B).

Complementing these findings, early X-ray crystallography studies by MacRae et al. on hAgo2 bound to guide RNA revealed a critical kink between nucleotides 6 and 7 of the seed region [45]. This kink is induced by the intercalation of Ile365 from α-helix 7, disrupting the A-form helical stacking of the guide RNA. Upon target RNA binding, helix 7 shifts approximately 4 Å to interact with the minor groove of the guide-target duplex, relieving the kink and restoring the full A-form helix, facilitating optimal base pairing beyond position 5. This movement is essential for target RNA recognition and duplex stabilization, as it generates a minor groove that allows helix 7 to stabilize the open conformation of the guide RNA. In line with these structural insights, Richter et al. further emphasized the critical role of base pairing dynamics at the junction of the seed and central regions in maintaining target specificity [32]. The introduction of neutral backbone modifications, such as MOP and iBu, can disrupt this delicate mechanism. MD simulations show that such modifications could induce structural changes in the phosphate backbone, leading to a kink at positions 6–7 and altered interactions between the guide RNA and hAgo2. These modifications may prevent helix-7 from adopting the proper conformation, potentially distorting the minor groove and hindering the target recognition process. For instance, the MOP backbone introduces a greater kink to accommodate the methoxypropyl fragment within the PIWI binding pocket, as evidenced by changes in backbone angles. This suggests that modifications in the seed region could perturb the interaction between the guide and target RNAs. Consequently, this disruption could lead to the misalignment of helix 7, impeding efficient target pairing and perhaps promoting the displacement of partially complementary off-target RNAs.

Overall, these findings highlight the fine-tuned nature of hAgo2-guide RNA interactions and suggest that even subtle changes in the backbone can significantly alter the efficiency and specificity of target recognition, with potential implications for both RNA interference and off-target effects in therapeutic applications.

### Stereochemical purity is not required for alkyl phosphonate-modified siRNAs to be effective

**Scheme 2.**
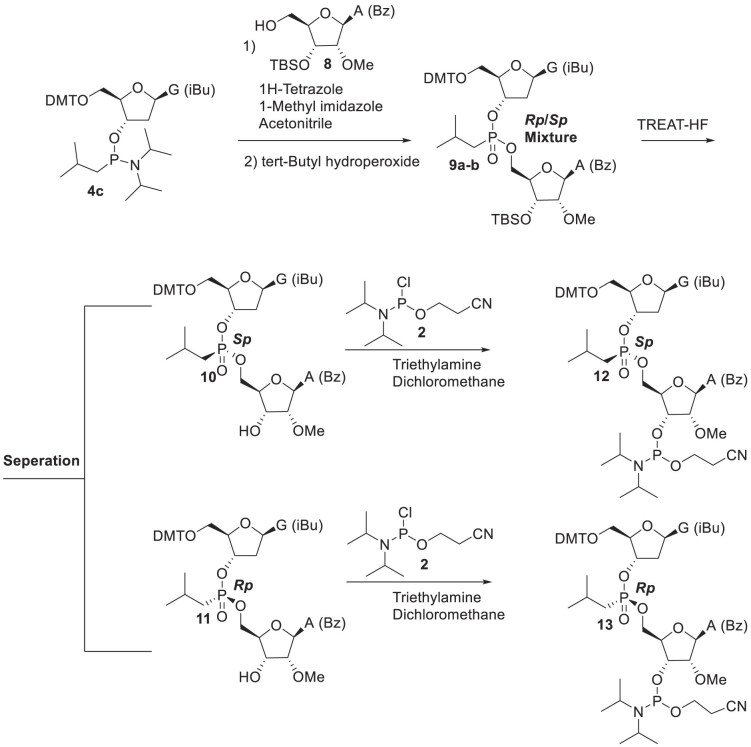
Synthesis of *Rp* and *Sp* A-G dimer phosphoramidite **12** and **13**. DMT: 4,4**′**-dimethoxytrityl; iBu: isobutyryl; Bz: benzoyl; TBS: tert-butyldimethylsilyl.

**Figure 14.**
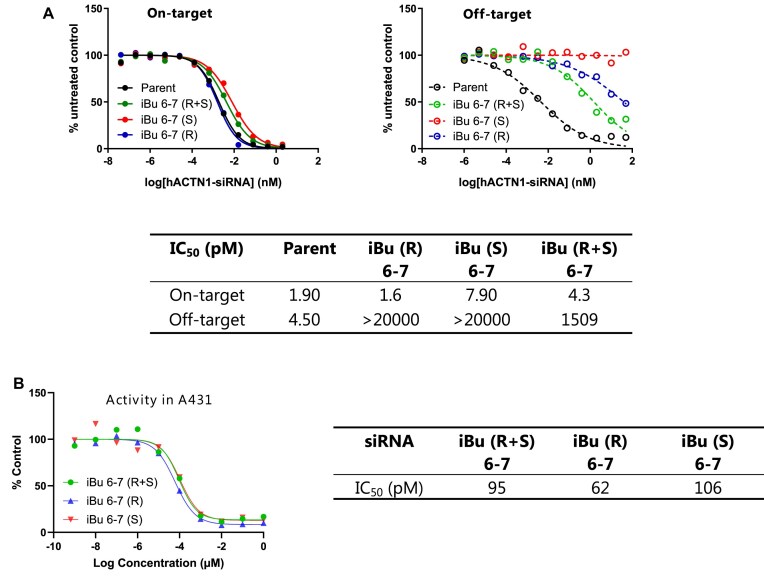
(**A**) Dual-luciferase reporter assay results for *ACTN1* siRNAs modified with a single *Rp* or *Sp* iBu at positions 6–7 of the guide strand. Solid line: on-target; dashed line: off-target. (**B**) *In vitro* activities of the *ACTN1* siRNAs modified with a single *Rp* or *Sp* iBu at positions 6–7 in A431 cell line (lipid transfection, 96 h),

During the synthesis of alkyl phosphonates, much like phosphorothioates, a chiral center at the phosphorus atom is formed, resulting in a mixture of *Rp* and *Sp* diastereomers. Throughout these studies, we chose not to separate these diastereomers, as the *Rp*/*Sp* mixture effectively mitigated siRNA off-target effects. However, we were interested in understanding how chirality impacts their function. To investigate this, we selected iBu as a representative modification and incorporated it at positions 6–7 of the *ACTN1* guide strand (refer to Table 1, line 6) using pre-synthesized *Rp* and *Sp* dimers, as shown in Scheme 2 and explained in the Materials and Methods section. Briefly, guanosine phosphonamidite **4c** was reacted with N-protected-2′-*O*-methyl-3′-*O*- tert-butyldimethylsilyl adenosine **8** to form the *Rp* and *Sp* A–G dimers **9a-b**. The tert-butyldimethylsilyl (TBS) group was then removed using triethylamine trihydrofluoride (TREAT-HF), and the pure diastereomers **10** and **11** were separated by reverse-phase chromatography. Their identities were confirmed by ³¹P NMR and distinct *R*_f_ values on TLC. Each isomer was then converted to the corresponding phosphoramidites to yield compounds **12** and **13**. The dimers were incorporated into the *ACTN1* guide strand using standard procedures and assessed for on-target and off-target activity using the dual-luciferase assay. Both isomers effectively mitigated off-target effects. The *Sp* isomer provided slightly improved off-target reduction with a modest trade-off in on-target potency, while the *Rp* isomer showed slightly better on-target activity with reduced off-target effects (Fig. 14A–B). As noted in previous sections, the dual-luciferase assay lacks the precision required for absolute IC_50_ determination. To obtain a more precise assessment of IC_50_ values, we evaluated these two siRNAs along with the *Rp*/*Sp* mixture in the A431 cell line. Consistent with the dual-luciferase assay, the *Rp* isomer showed a slightly more favorable IC_50_; however, the difference was not substantial. Taken together, these complementary activity profiles indicate that the use of the diastereomeric mixture is both practical and efficient under the conditions tested. Given the marginal differences observed between the individual isomers in this context, the increased synthetic complexity and resource investment required for stereoisomer separation may not be warranted for most applications. However, it remains possible that specific sequence contexts or therapeutic targets could benefit from stereopure compounds. Thus, the isomeric mixture currently represents a streamlined and practical approach for siRNA applications.

## Discussion

The present study demonstrates the potential of siRNAs modified with single alkyl phosphonate linkages to improve the therapeutic profile of RNA interference-based treatments. Our findings highlight several key insights regarding the optimization of siRNA backbone modifications for enhanced specificity and efficacy. A particularly noteworthy finding was that the modification at the internucleotide linkage between positions 6–7 from the 5′-end of the guide strands proved to be the most effective. This “sweet spot” for modification was consistent across different siRNA sequences, as shown with *Ttr*, *Hao1*, *ACTN1*, and *Marc1* siRNAs. While modifications at positions 5–7 in the seed region are typical for off-target mitigation, our study highlights the novel use of backbone modifications at the 6–7 internucleotide linkage. This suggests that backbone modifications may influence siRNA functionality differently from nucleotide modifications. While further investigation is needed, our data suggest that the modifications at positions 6–7 likely induce local conformational changes that enhance siRNA selectivity without disrupting on-target activity.

Among the alkyl phosphonate modifications tested, isobutyl (iBu) phosphonate emerged as the most effective in balancing off-target mitigation and on-target activity. However, we cannot rule out the potential of smaller alkyl chains, which may also offer effective modifications. Although larger modifications, such as MOP, showed comparable off-target effects, they negatively impacted on-target activity, indicating that longer carbon chains may interfere with siRNA efficacy. These findings underscore the importance of fine-tuning backbone chemistry to optimize both specificity and functionality. The consistency between *in vitro* dual luciferase assay results and *in vivo* findings strengthens the validity of our screening approach. While the use of dual luciferase assays for screening has been previously reported, relatively few studies have successfully demonstrated *in vivo* translatability. Our findings contribute to this growing field by showing that the modified siRNAs retained their activity in both settings, with *in vitro* results correlating well with *in vivo* efficacy. This suggests that the screening method employed here is reliable for identifying siRNA candidates that perform well in early-stage experimental models.

DGE analysis revealed significant off-target mitigation. Additionally, the modified siRNAs exhibited an enhanced safety profile *in vivo*, as evidenced by reduced ALT/AST levels and the absence of histopathological changes in treated mice. These findings reinforce the connection between off-target mitigation and reduced toxicity markers, aligning with prior observations that minimizing off-target effects, particularly in the liver, can enhance safety. Collectively, these results suggest that alkyl phosphonate modifications could mitigate adverse effects, thereby improving the clinical potential of siRNA-based treatments. While these results are promising, it is important to recognize certain limitations of this study. Our research focused on a limited number of target genes and siRNA sequences, which may affect the generalizability of the findings. Future studies should aim to explore a broader range of siRNA sequences and gene targets to validate whether the 6–7 position modification is universally effective. Additionally, long-term safety and efficacy studies in animal models will be essential to comprehensively evaluate the clinical potential of these modified siRNAs. In summary, our study suggests that alkyl phosphonate modifications, particularly at positions 6–7, represent a novel and effective approach to improving siRNA specificity and safety. As the field of siRNA therapeutics continues to advance, these precisely engineered backbone modifications could play a critical role in the development of safer, more effective RNA interference treatments for a wide range of diseases. Future work should continue to optimize these modifications and explore their potential in clinical settings.

## Supplementary Material

gkaf692_Supplemental_File

## Data Availability

The RNA-seq data reported in this article have been deposited in NCBI’s Gene Expression Omnibus (GEO) and are accessible through GEO Series accession number GSE285764.
