# Supplementary material for: Single alkyl phosphonate modification of the siRNA backbone in the seed region enhances specificity and therapeutic profile
Source: Nucleic Acids Res. 2025 Jul 30;53(14):gkaf692. doi: 10.1093/nar/gkaf692 (PMC12309379; doi:10.1093/nar/gkaf692)
Supplement: gkaf692_Supplemental_File [file gkaf692_supplemental_file.docx]

**­Supplementary Data**

**­Single Alkyl Phosphonate Modification of the siRNA Backbone in the Seed Region Enhances Specificity and Therapeutic Profile**

Mehran Nikan *, Qingfeng Li, Michael Tanowitz, Hongda Li, Sagar Damle, Marie Annoual, Rodrigo Galindo-Murillo, Audrey Low, Stephanie Klein, Clare Quirk, Guillermo Vasquez, W. Brad Wan, Andrew T. Watt, Michael T. Migawa, Eric E. Swayze, Thazha P. Prakash *

Ionis Pharmaceuticals Inc., 2855 Gazelle Ct, Carlsbad, CA 92010, USA

* To whom correspondence should be addressed. Tel: +1 760 603 2568; Email: mnikan@ionis.com

Correspondence may also be addressed to Thazha P. Prakash. Email: TPrakash@ionis.com

**List of contents**

**Table S1 S2**

Figure S1 S3

Figure S2 S4

Figure S3 S5

Figures S4-S21 (NMR Spectra) S6-S23

| **Target** | **As/**  **Sense** | **Backbone**  **Modification** | **Formula** | **Average Mass** | **Observed Mass** | **UV purity (%)** |
| --- | --- | --- | --- | --- | --- | --- |
| *Ttr* | Sense | N/A | C_280_H_390_N_81_O_171_P_21_S_2_F_4_ | 8417.0 | 8416.0 | 96.0 |
| *Ttr* | As | None | C_242_H_310_N_85_O_154_P_23_S_4_F_4_ | 7790.1 | 7789.2 | 95.0 |
| *Ttr* | As | dA5  MOP 5-6 | C_245_H_316_N_85_O_153_P_23_S_4_F_4_ | 7816.2 | 7815.6 | 93.1 |
| *Ttr* | As | dG6  MOP 6-7 | C_246_H_319_N_85_O_154_P_23_S_4_F_3_ | 7828.2 | 7827.6 | 95.9 |
| *Ttr* | As | dA5  MP 5-6 | C_242_H_310_N_85_O_152_P_23_S_4_F_4_ | 7758.1 | 7757.2 | 94.1 |
| *Ttr* | As | dG6  MP 6-7 | C_243_H_313_N_85_O_153_P_23_S_4_F_3_ | 7770.2 | 7769.6 | 93.5 |
| *Ttr* | As | dA7  MP 7-8 | C_242_H_310_N_85_O_152_P_23_S_4_F_4_ | 7758.1 | 7757.2 | 91.0 |
| *Ttr* | As | dG6  Prop 6-7 | C_245_H_317_N_85_O_153_P_23_S_4_F_3_ | 7798.2 | 7797.6 | 90.5 |
| *Ttr* | As | dG6  iBu 6-7 | C_246_H_319_N_85_O_153_P_23_S_4_F_3_ | 7812.2 | 7811.2 | 95.5 |
| *Ttr* | As | dG6  cHex 6-7 | C_248_H_321_N_85_O_153_P_23_S_4_F_3_ | 7838.3 | 7837.6 | 95.4 |
| *Ttr* | As | 2´-F G6  MOP 6-7 | C_246_H_318_N_85_O_154_P_23_S_4_F_4_ | 7846.2 | 7745.6 | 95.2 |
| *Ttr* | As | 2´-OMe G6  MOP 6-7 | C_247_H_321_N_85_O_155_P_23_S_4_F_3_ | 7858.3 | 7857.1 | 93.7 |
| *Ttr* | As | dG6  PACE 6-7 | C_244_H_313_N_85_O_155_P_23_S_4_F_3_ | 7814.2 | 7813.2 | 94.8 |
| *ACTN1* | Sense | N/A | C_279_H_389_N_78_O_173_P_21_S_4_F_4_ | 8458.1 | 8457.1 | 96.5 |
| *ACTN1* | As | None | C_244_H_314_N_93_O_148_P_23_S_4_F_4_ | 7834.2 | 7833.6 | 95.4 |
| *ACTN1* | As | dT5  MOP 5-6 | C_248_H_322_N_93_O_147_P_23_S_4_F_4_ | 7874.3 | 7873.1 | 93.3 |
| *ACTN1* | As | dG6  MOP 6-7 | C_248_H_323_N_93_O_148_P_23_S_4_F_3_ | 7872.3 | 7871.2 | 92.4 |
| *ACTN1* | As | dT5  iBu 5-6 | C_248_H_322_N_93_O_146_P_23_S_4_F_4_ | 7858.3 | 7857.3 | 94.8 |
| *ACTN1* | As | dG6  iBu 6-7 | C_248_H_323_N_93_O_147_P_23_S_4_F_3_ | 7856.3 | 7855.6 | 93.8 |
| *Marc1* | Sense | N/A | C_281_H_392_N_85_O_170_P_21_S_2_F_4_ | 8471.1 | 8470.1 | 96.1 |
| *Marc1* | As | None | C_243_H_313_N_90_O_151_P_23_S_4_F_4_ | 7827.2 | 7826.2 | 94.6 |
| *Marc1* | As | dG6  iBu 6-7 | C_247_H_322_N_90_O_150_P_23_S_4_F_3_ | 7849.3 | 7848.4 | 93.6 |
| *Hao* | Sense | N/A | C_286_H_397_N_95_O_165_P_21_S_2_F_3_ | 8577.3 | 8576.4 | 93.1 |
| *Hao* | As | None | C_236_H_300_N_75_O_158_P_23_S_4_F_6_ | 7669.9 | 7668.2 | 94.5 |
| *Hao* | As | dG6  MOP 6-7 | C_240_H_311_N_75_O_158_P_23_S_4_F_5_ | 7710.0 | 7709.2 | 96.5 |
| *Hao* | As | 2´-F G6  MOP 6-7 | C_240_H_310_N_75_O_158_P_23_S_4_F_6_ | 7728.0 | 7727.2 | 96.0 |

Table S1. Mass and UV purity characterization of synthesized siRNA strands

| **siRNA** | **Modification** | **On-target**  **IC_50_ (pM)** | **Off-target**  **IC_50_ (pM)** |
| --- | --- | --- | --- |
| **5´-VP-TsUsAUAGAGCAAGAACACUGUUsUsU-3´**  **3´-GalNAc-AAUAUCUCGUUCUUGUGACsAsA-5´** | Parent | 0.34 | 13.3 |
| **5´-VP-TsUsAUAGpaceAGCAAGAACACUGUUsUsU-3´**  **3´-GalNAc-AAUAUCUCGUUCUUGUGACsAsA-5´** | dG6  PACE 6-7 | 0.47 | 28.1 |

Figure S1. Dual-Luciferase Reporter Assay results for *Ttr* siRNAs modified with dG at position 6 and PACE at 6-7. Solid line: on-target; dashed line: off-target.

| **siRNA** | **Modification** | **On-target**  **IC_50_ (pM)** | **Off-target**  **IC_50_ (pM)** |
| --- | --- | --- | --- |
| **5′-VP-TsUsGUCGAUGACUUUCACAUUCsUsG-3′**  **3´-GalNAc-AACAGCUACUGAAAGUGUAsAsG-5′** | Parent | 29 | 58 |
| **5′-VP-TsUsGUCGmopAUGACUUUCACAUUCsUsG-3′**  **3´-GalNAc-AACAGCUACUGAAAGUGUAsAsG-5′** | dG6  MOP 6-7 | 4.0 | 641 |
| **5′-VP-TsUsGUCGmopAUGACUUUCACAUUCsUsG-3′**  **3´-GalNAc-AACAGCUACUGAAAGUGUAsAsG-5** | 2’-F G6  MOP 6-7 | 2.7 | 438 |

Figure S2. Dual-Luciferase Reporter Assay results for *Hao1* siRNAs modified with dG and 2**´-**F G at position 6, and MOP at 6-7. Solid line: on-target; dashed line: off-target. Details of the assay design are described in the Materials and Methods section. The on-target reporter vector for *Hao1* contained a single fully complementary site to the guide strand of the *Hao1*, inserted into the 3´-untranslated region (3´ UTR) of the Renilla luciferase cassette. The target site sequence was 5´-CAGAATGTGAAAGTCATCGACAA-3´. The off-target reporter plasmid for *Hao1* contained four seed-complementary sites separated by a 19-nucleotide spacer sequence, also inserted into the 3´ UTR of the Renilla cassette. The seed-complementary sequence was 5´-CATCGACAA-3´, with the spacer sequence 5´-TAATATTACTTAAATATAA-3´.


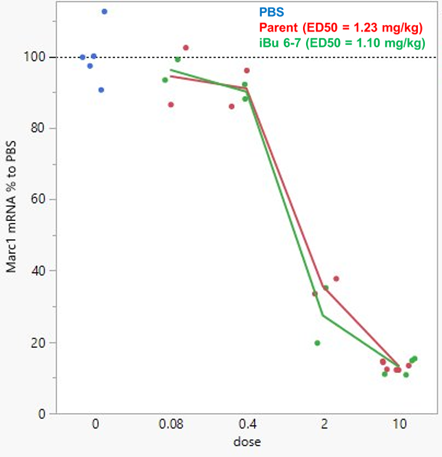


Figure S3. In vivo activities of parent and iBu-modified *Marc1* siRNAs in mice. (0.08, 0.4, 2, 10 mg/kg, 3 days).

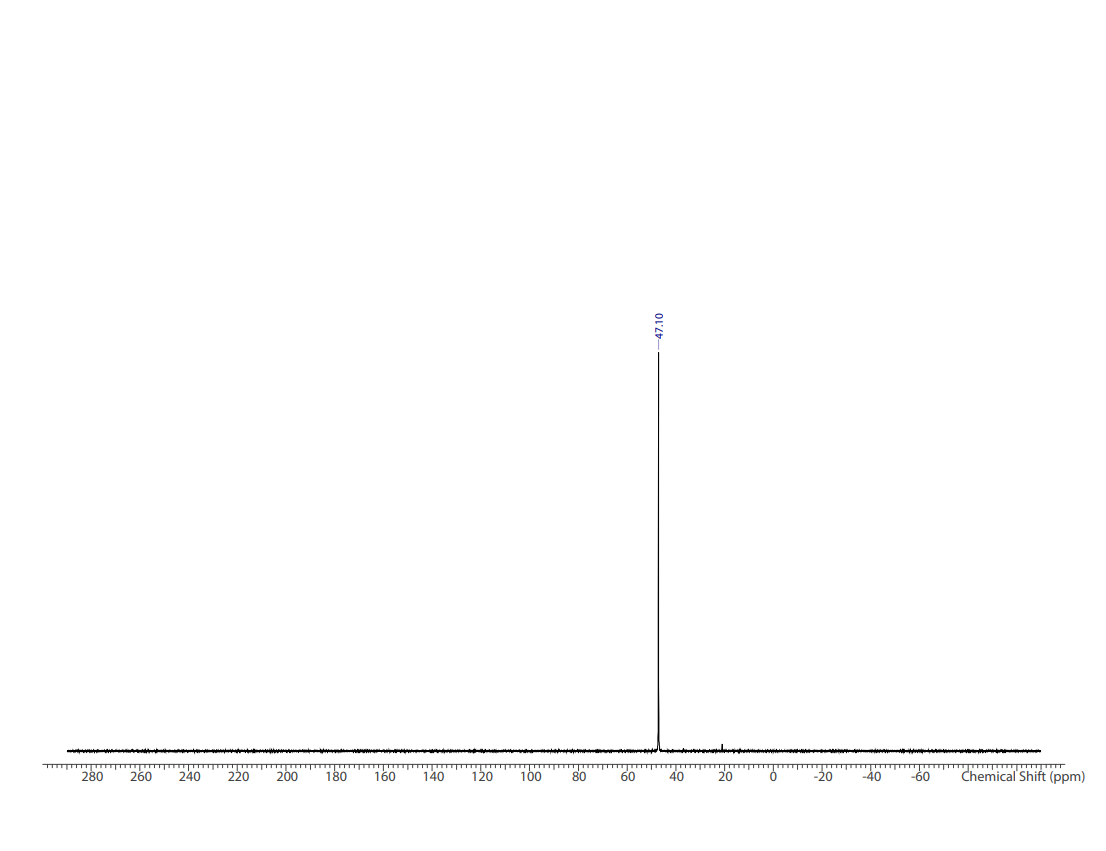


Figure S4. ^31^P NMR spectrum of Propyl-bis(diisopropylamino)phosphine **3d** (CDCl_3_)


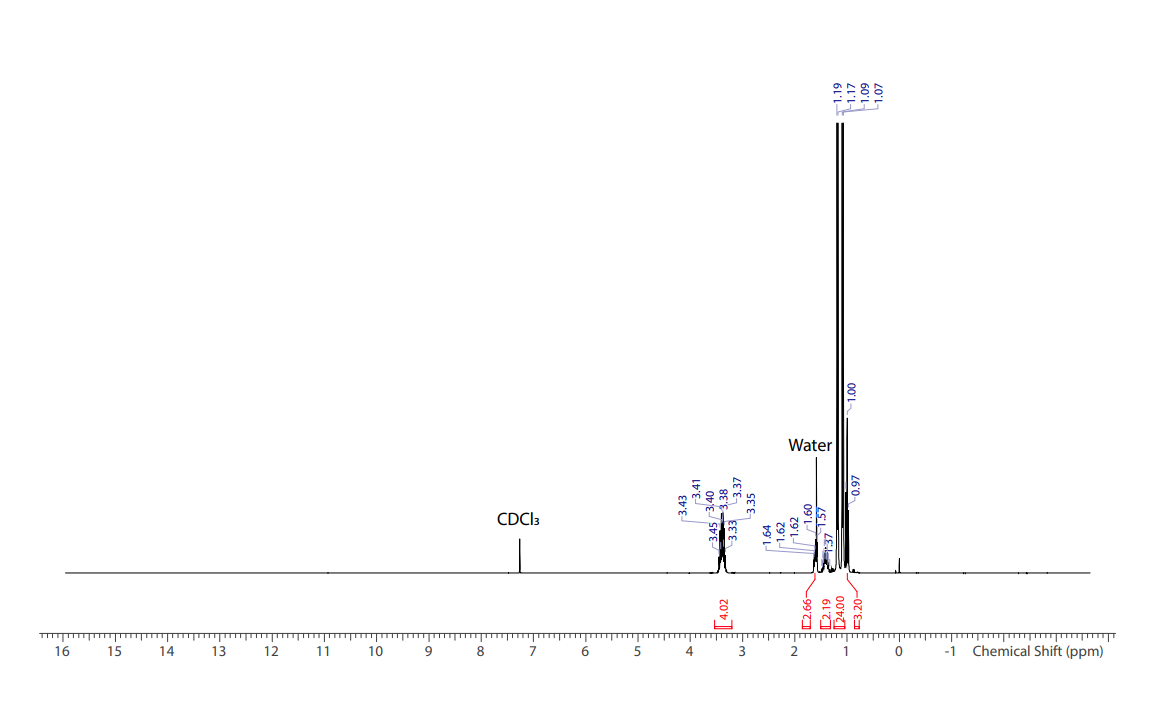


Figure S5. ^1^H NMR spectrum of Propyl-bis(diisopropylamino)phosphine **3d** (CDCl_3_)


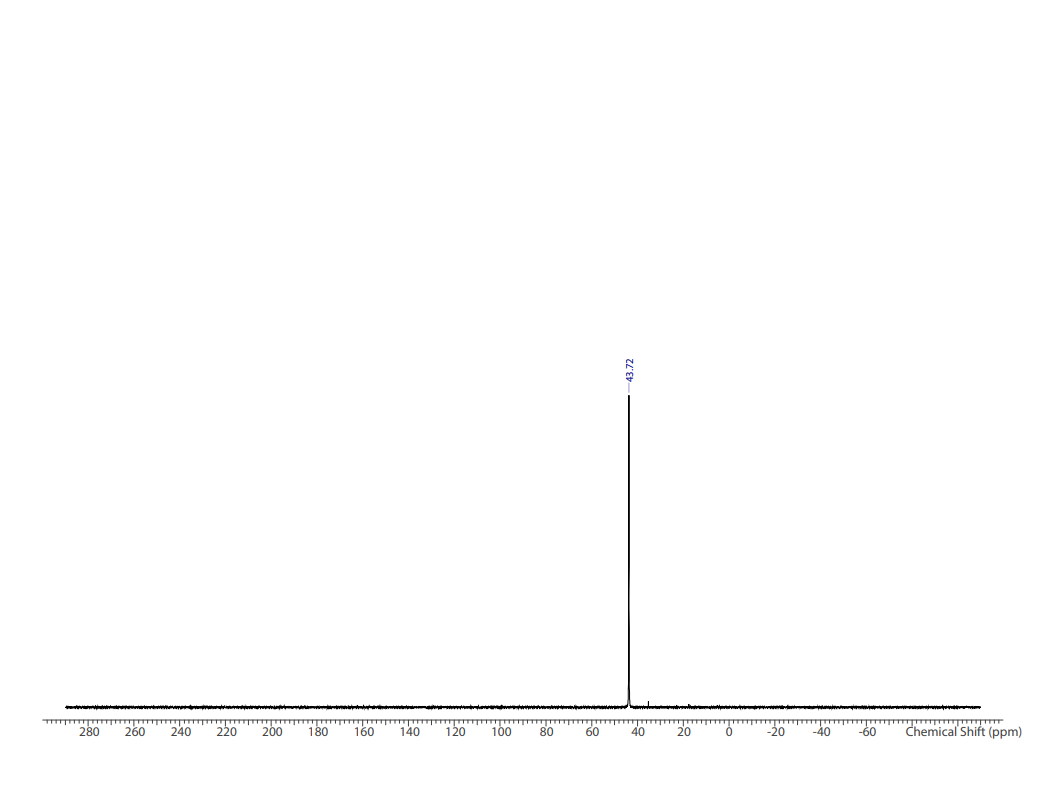


Figure S6. ^31^P NMR spectrum of Isobutyl-bis(diisopropylamino)phosphine **3c** (acetone-d6)


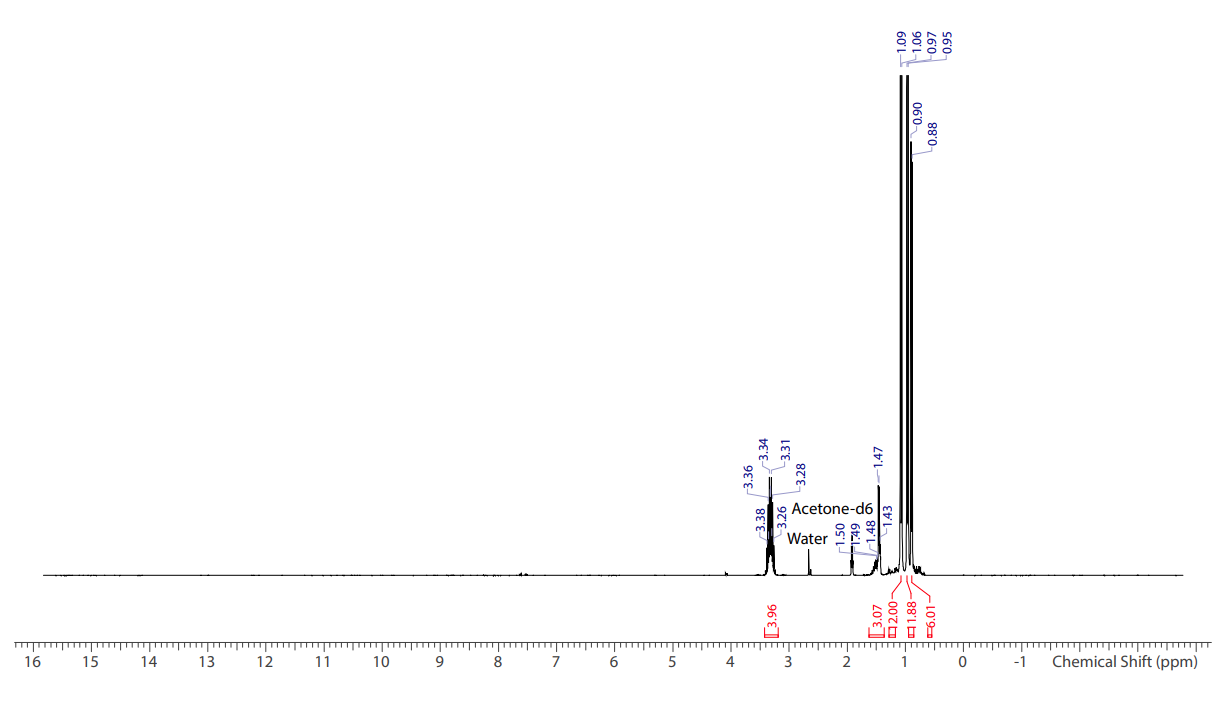
Figure S7. ^1^H NMR spectrum of Isobutyl-bis(diisopropylamino)phosphine **3c** (acetone-d6)


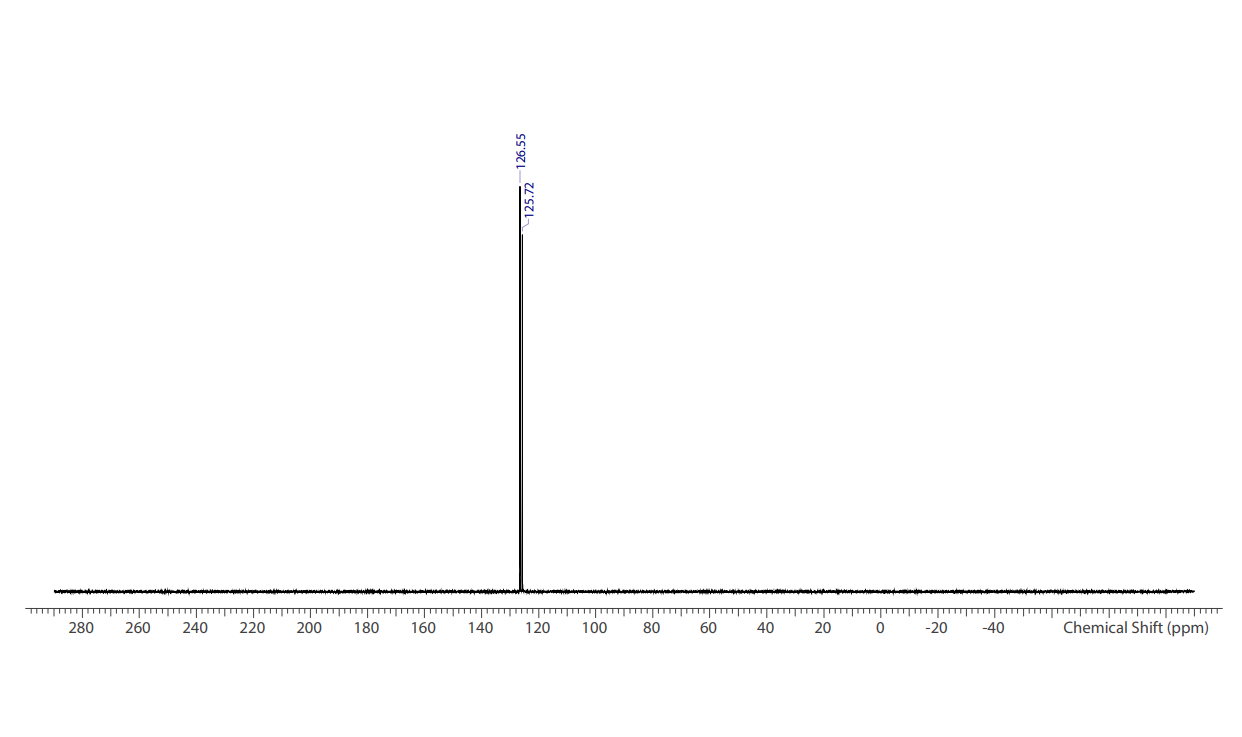


Figure S8. ^31^P NMR spectrum of Propyl Phosphonamidite **4d** (acetone-d6)


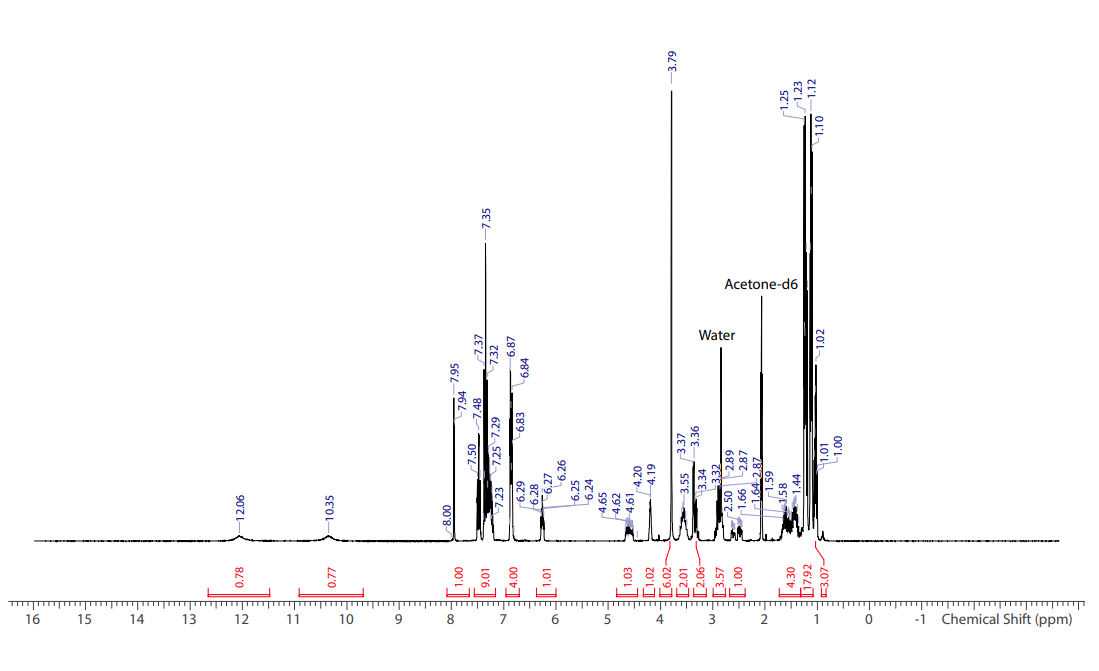
Figure S9. ^1^H NMR spectrum of Propyl Phosphonamidite **4d** (acetone-d6)

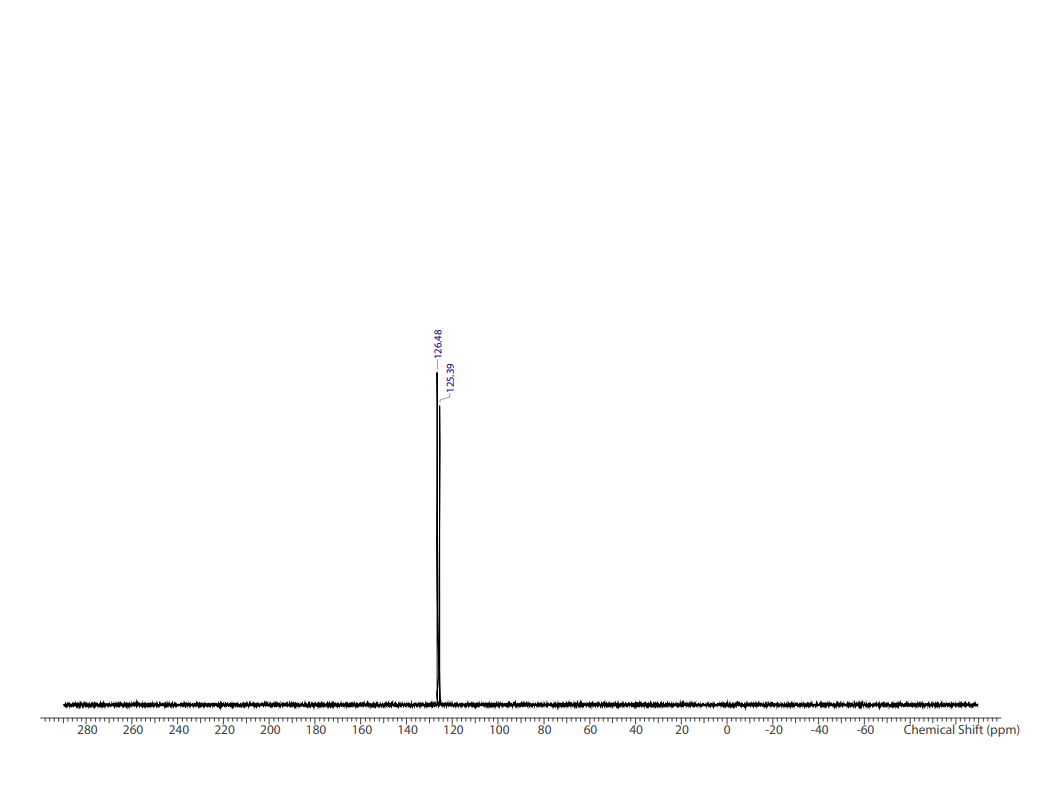


Figure S10. ^31^P NMR spectrum of Isobutyl Phosphonamidite **4c** (CDCl_3_)


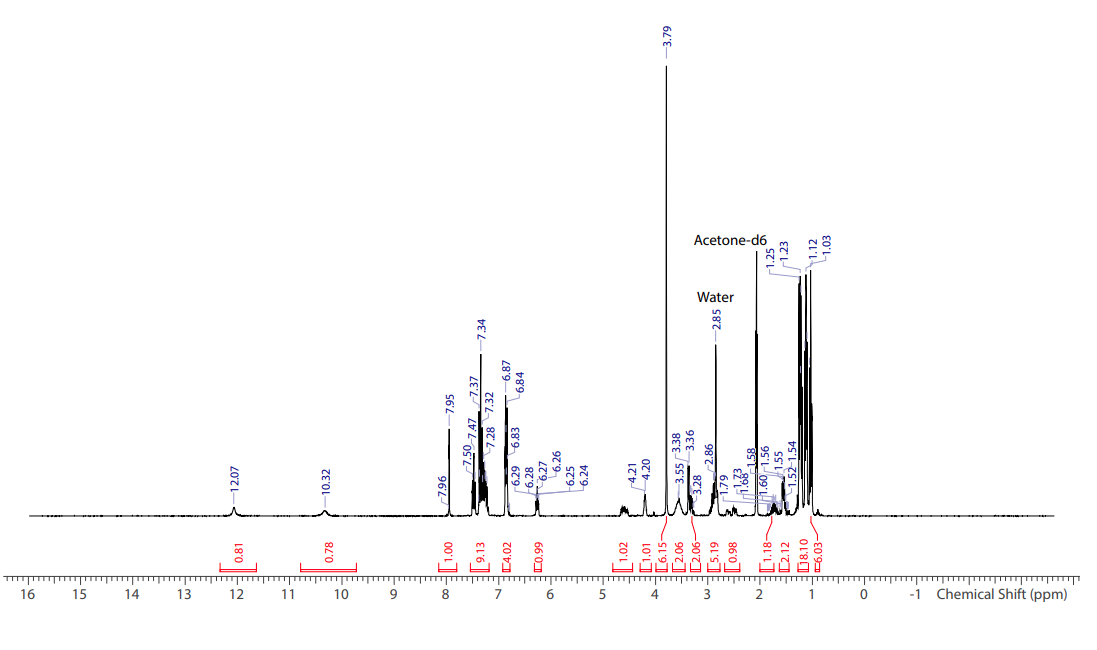
Figure S11. ^1^H NMR spectrum of Isobutyl Phosphonamidite **4c** (acetone-d6)


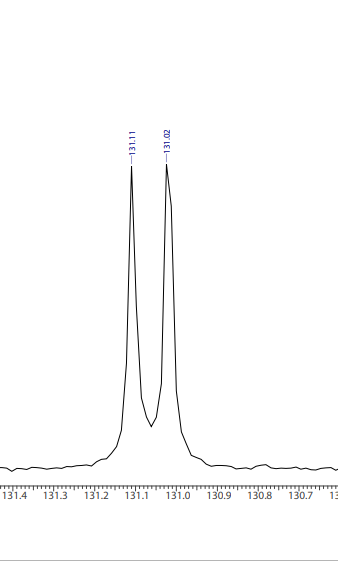

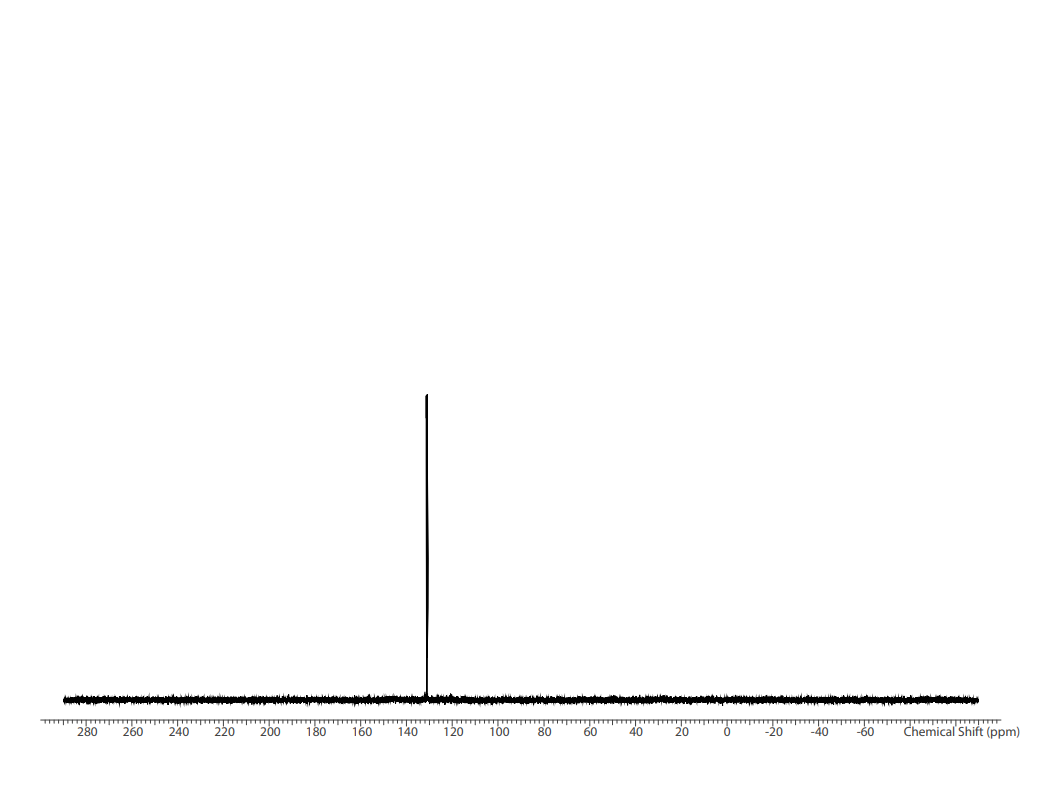


Figure S12. ^31^P NMR spectrum of Cyclohexyl Phosphonamidite **4a** (CDCl_3_)


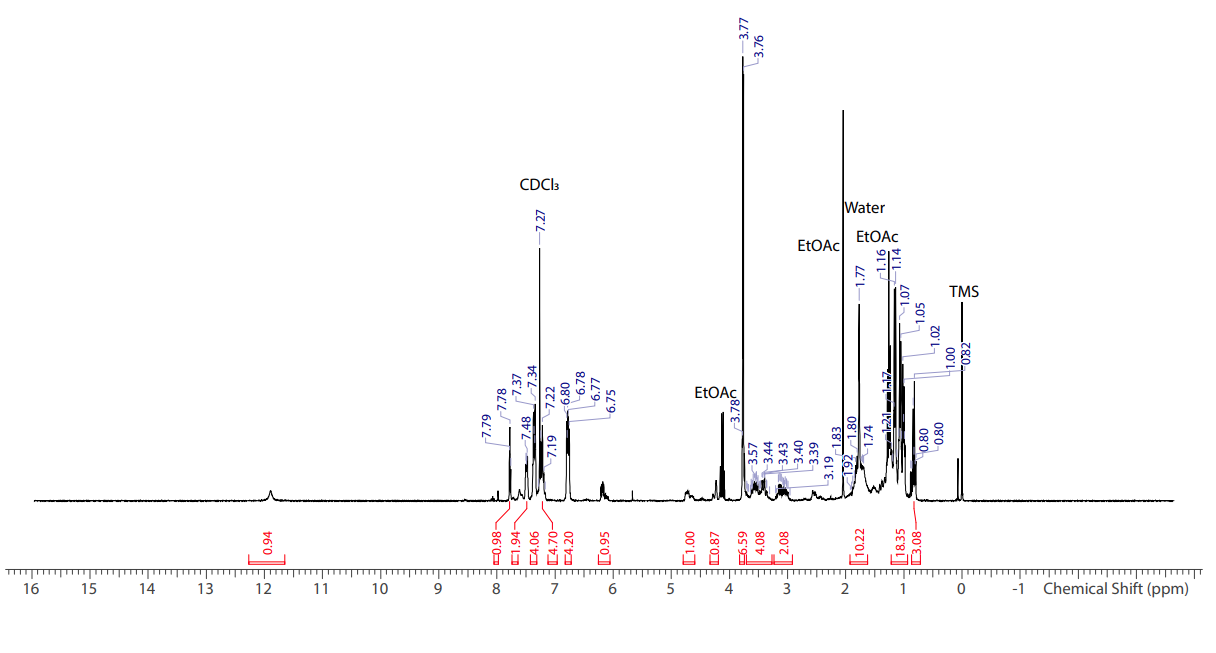


Figure S13. ^1^H NMR spectrum of Cyclohexyl Phosphonamidite **4a** (CDCl_3_).


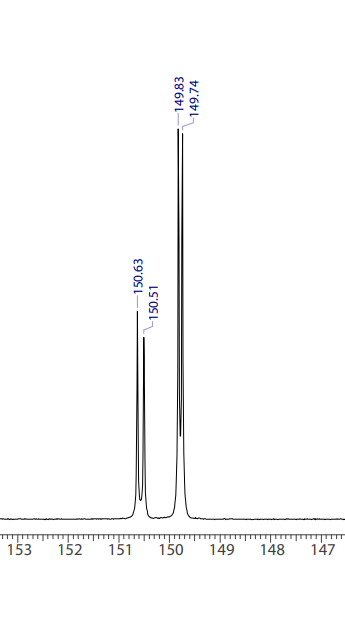

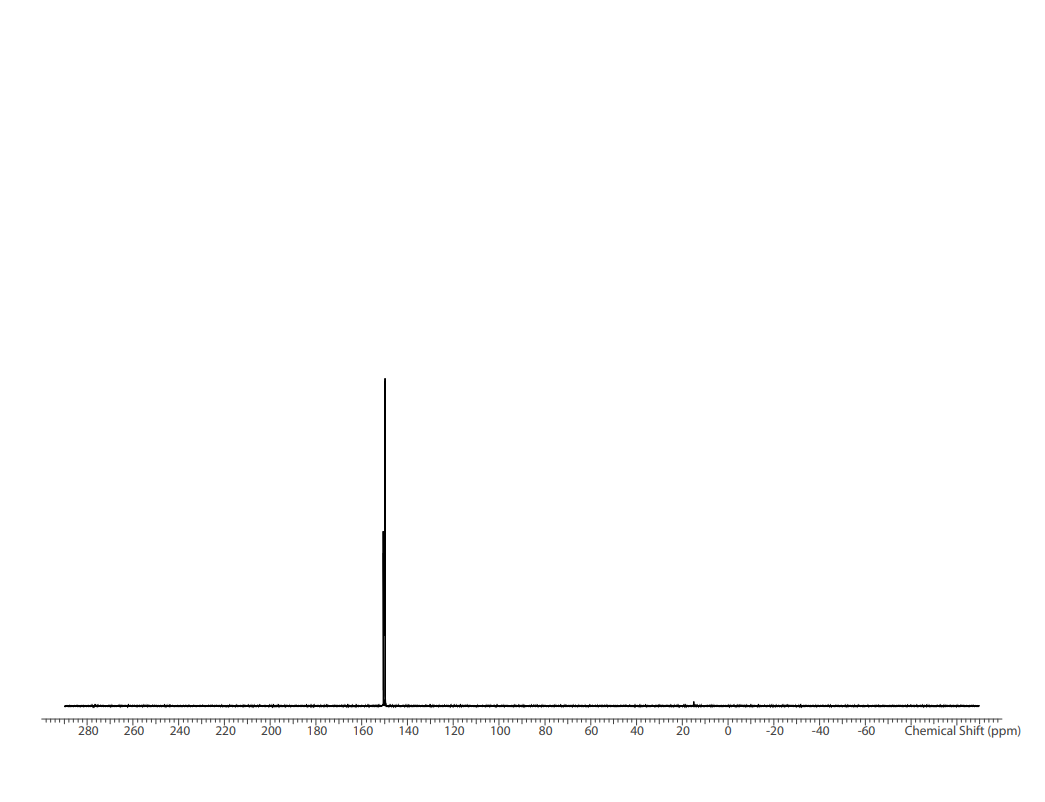


Figure S14. ^31^P NMR spectrum of 2´-F G phosphonamidite **6b** (CDCl_3_)


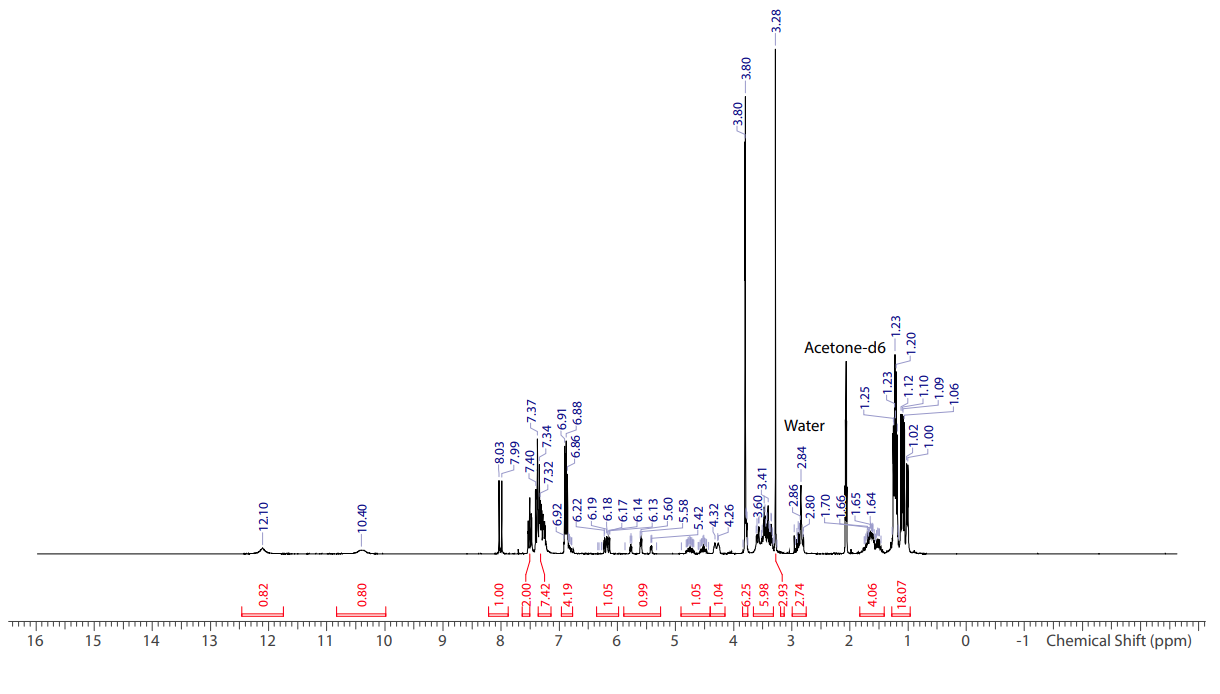


Figure S15. ^1^H NMR spectrum of 2´-F G phosphonamidite **6b** (acetone-d6)

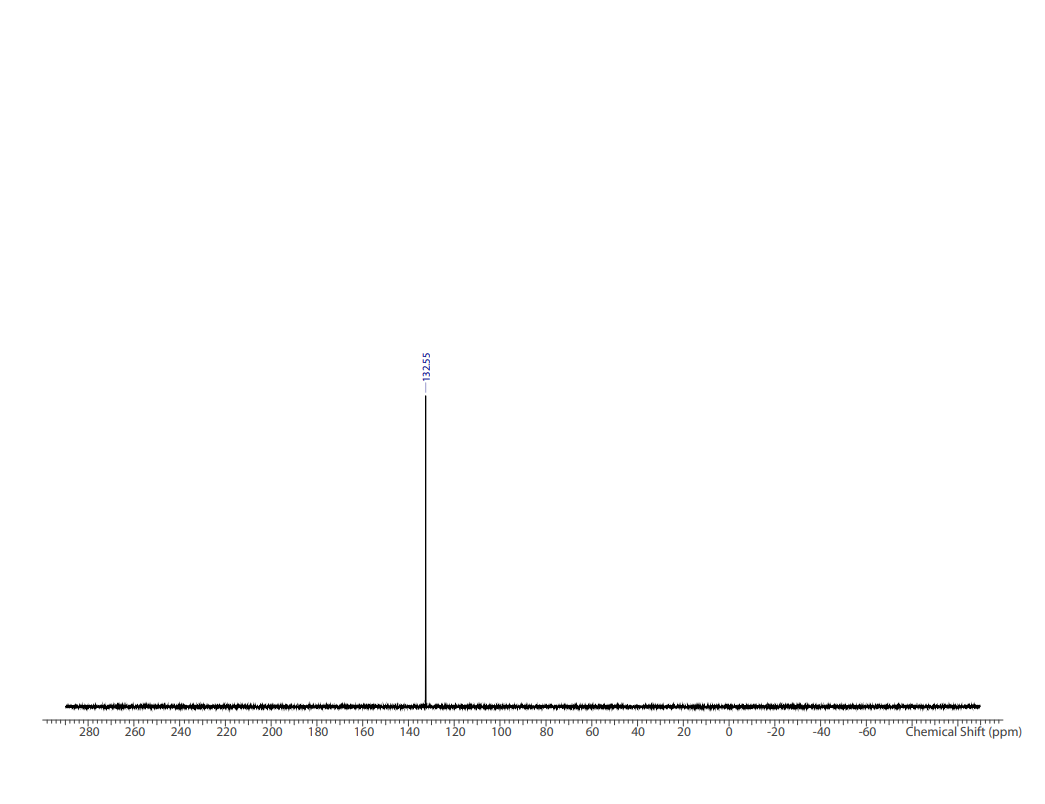


Figure S16. ^31^P NMR spectrum of 2´-OMe G phosphonamidite **7b** (CDCl_3_)


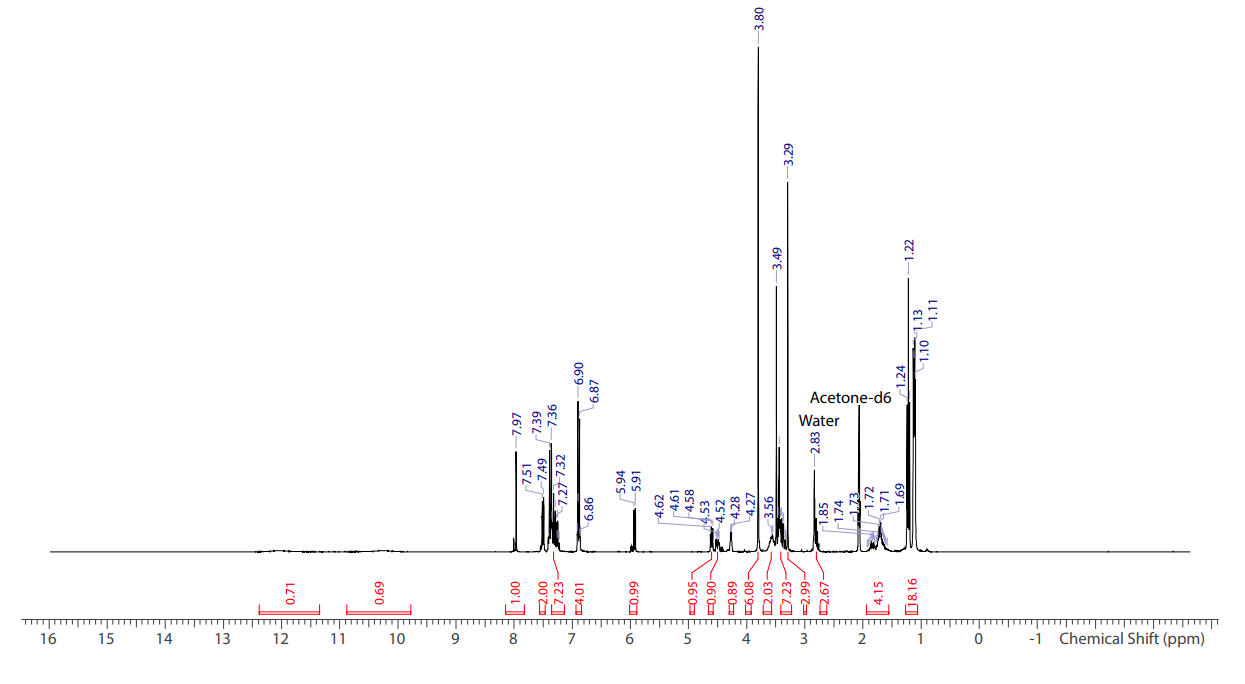


Figure S17. ^1^H NMR spectrum of 2´-OMe G phosphonamidite **7b** (acetone-d6)


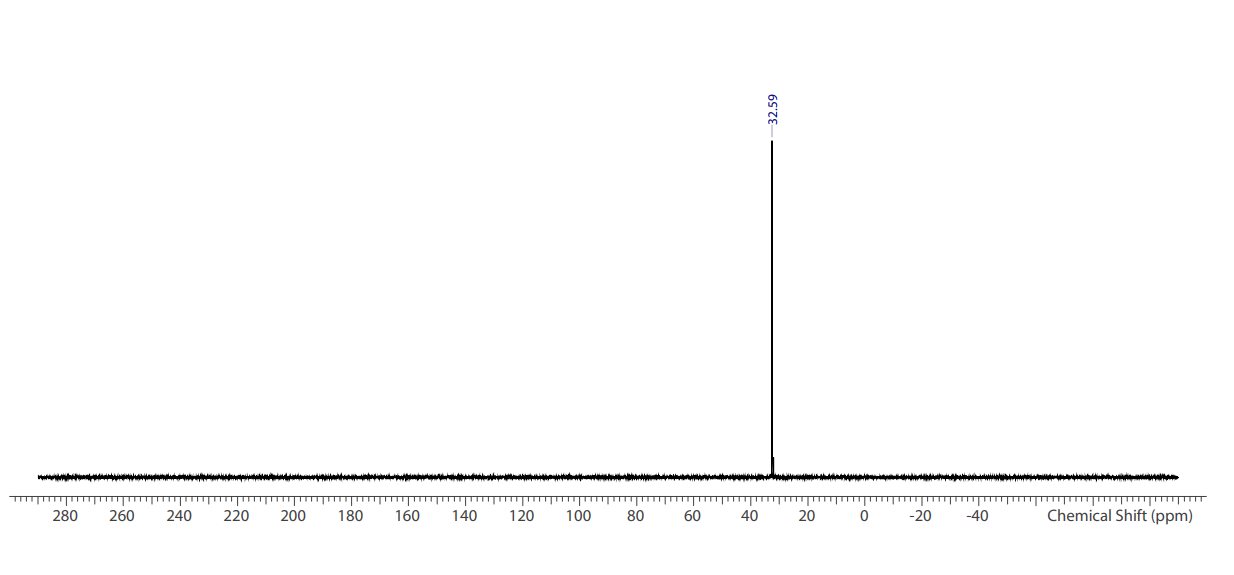


Figure S18. ^31^P NMR spectrum of *Rp* A–G dimers **10** (acetone-d6)

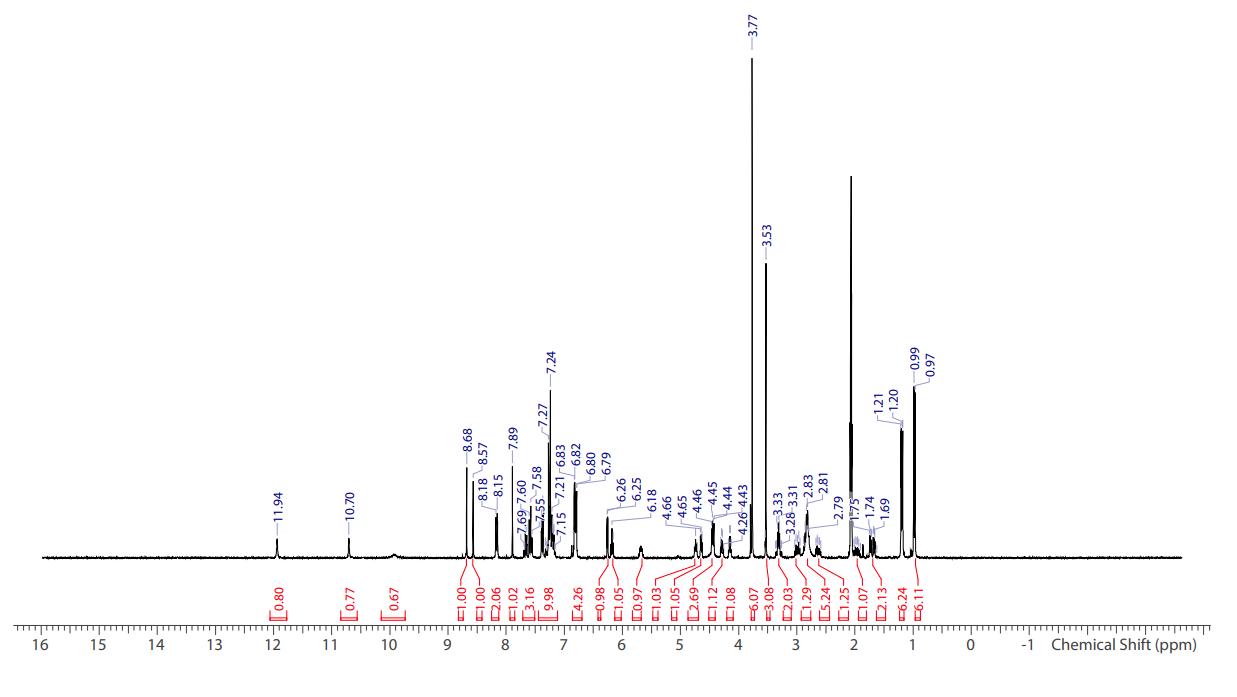
Figure S19. ^1^H NMR spectrum of *Rp* A–G dimers **10** (acetone-d6)


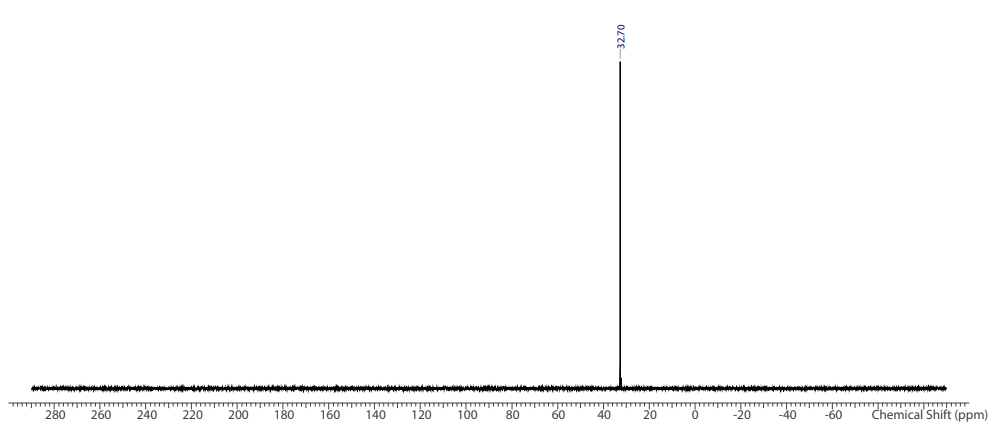
Figure S20. ^31^P NMR spectrum of *Rp* A–G dimers **11** (acetone-d6)


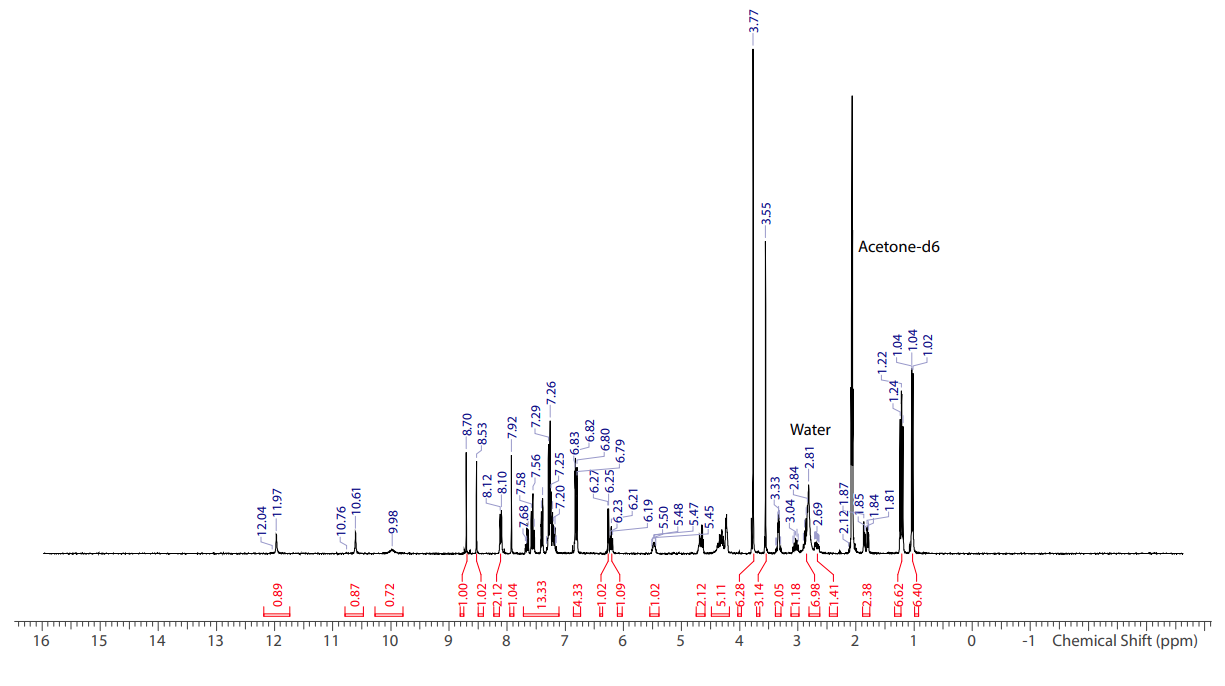


Figure S21. ^1^H NMR spectrum of *Rp* A–G dimers **11** (acetone-d6)
